# Space‐use by feral cattle and horses shapes vegetation structure in a trophic rewilding area

**DOI:** 10.1002/eap.70170

**Published:** 2026-02-04

**Authors:** Jeppe Å. Kristensen, Robert Buitenwerf, Emilio Berti, Oskar L. P. Hansen, Simon D. Schowanek, Rasmus Ejrnæs, Morten D. D. Hansen, Kent Olsen, Signe Normand, Jens‐Christian Svenning

**Affiliations:** ^1^ Center for Ecological Dynamics in a Novel Biosphere (ECONOVO), Department of Biology Aarhus University Aarhus C Denmark; ^2^ Center for Sustainable Landscapes Under Global Change (SustainScapes) Department of Biology, Aarhus University Aarhus C Denmark; ^3^ Section for Ecoinformatics and Biodiversity Department of Biology, Aarhus University Aarhus C Denmark; ^4^ Leverhulme Centre for Nature Recovery, School of Geography and the Environment, University of Oxford Oxford UK; ^5^ German Centre for Integrative Biodiversity Research (iDiv) Leipzig Germany; ^6^ Institute of Biodiversity, Friedrich­Schiller­University Jena Jena Germany; ^7^ Department of Research and Collections Natural History Museum Aarhus Aarhus C Denmark; ^8^ Department of Ecoscience Aarhus University Aarhus C Denmark

**Keywords:** drought, feral cattle, feral horses, large herbivores, NDVI, shrub encroachment, temperate Europe, trophic rewilding

## Abstract

Feral cattle (*Bos taurus*) and horses (*Equus ferus caballus*) are commonly introduced to European rewilding areas to halt vegetation succession and to conserve light‐demanding species. Yet, we still do not understand how the habitat preference of animals shapes vegetation structure at the landscape scale. Here, we used spatial preference modeling to understand drivers of space‐use based on GPS‐collared horses and cattle in a 120‐ha rewilding area in Denmark. Using a time series of a satellite‐based vegetation productivity index, we tested the ability of animal space‐use to explain changes in vegetation, as well as the trend of its spatial variability at the reserve scale, as a measure of landscape‐scale vegetation heterogeneity. We expected that animal space‐use would be driven mainly by topography and vegetation characteristics and that highly used areas with open vegetation would remain open. We, indeed, found that vegetation density and landscape connectivity were good predictors of space‐use preference for both cattle and horses. Additionally, both cattle and horses were strongly attracted to an artificial shelter located inside the reserve, warranting consideration of the use and placement of artificial infrastructure. Space‐use diverged during periods of resource scarcity emphasizing the value of introducing a variety of herbivore functional types for optimizing structural ecosystem heterogeneity. As expected, we found that cattle and horses slow down vegetation succession in highly used areas, as shown by the negative correlation between changes in growing season productivity and intensively used areas dominated by short herbaceous and shrubby vegetation. We could also show that the highly used areas showed the largest reductions and the fastest recovery in vegetation greenness following the pan‐European drought in 2018. A ~2/3 reduction in herbivore population size subsequent to the drought was followed by a general greening of the landscape, but with no clear relationship with space‐use intensity. Our study supports that trophic rewilding with year‐round grazing can limit vegetation densification at the landscape scale under near‐natural conditions. This is pertinent in the face of accelerating succession toward increasingly dark and tree‐dominated vegetation in temperate Europe's natural areas, and the associated biodiversity loss.

## INTRODUCTION

European ungulate communities hold only, on average, around 20% of the functional diversity and only 5% of the herbivore community biomass compared to the last interglacial, that is, before widespread human‐caused megafauna extinctions, with widespread negative consequences for particularly low‐stature light‐demanding biodiversity (Davoli et al., 2024; Svenning, Lemoine, et al., 2024). To mitigate this loss, feral cattle (*Bos taurus*) and horses (*Equus ferus caballus*) are often introduced as functional substitutes for the extinct conspecific original wild forms, aurochs (*Bos taurus primigenius*) and wild horses (*Equus ferus*), in trophic rewilding projects across Europe (Cromsigt et al., 2018). Trophic rewilding promotes self‐regulating biodiverse ecosystems by restoring trophic complexity (Svenning et al., 2016), and ecological studies in rewilding areas have shown that ungulate reintroduction can have long‐lasting positive effects on biodiversity (Ratajczak et al., 2022) that are dissimilar to conventional grazing with domestic animals (Allred et al., 2011; Kleppel & Frank, 2022; Mutillod et al., 2024). Despite theoretical and empirical evidence suggesting a positive effect of trophic rewilding on ecosystems, we still do not fully understand how introduced grazers use the landscape and how this can affect rewilding efforts. The vegetation structure and composition typically guide habitat preferences of herbivores (Klich et al., 2023; Pratt et al., 1986; Rech et al., 2025; Smagol et al., 2022). Both cattle and horses are generally perceived as obligate grassland species, but when given the opportunity, both species also use woody‐dominated habitats (Popp & Scheibe, 2014; Pratt et al., 1986). For example, a study from Germany showed that although horses and cattle obtained most of their forage from open pastures, horses spent 47% of their time in closed‐canopy forests compared to 37% for cattle (Popp & Scheibe, 2014). Typically, horses use a greater proportion of the available habitat space compared to cattle throughout the year, though seasonal divergence in space‐use is also observed (Rech et al., 2025). Direct competition for forage is rarely observed in natural systems but can occur during periods of resource scarcity (Clauss et al., 2003; Janis, 1976; Scasta et al., 2016). During scarcity, both horses and cattle supplement their herb‐dominated diet with woody biomass, but not necessarily the same type (Cromsigt et al., 2018; Hagstrup et al., 2021; Klich, 2017; Putman et al., 1987; Thomassen et al., 2023). For example, recent data from the study area of this paper showed that horses maintained a diverse diet year‐round with significant proportions of graminoids and deciduous trees, while cattle fed more selectively on forbs during summer and shrubs (particularly *Rubus*) during winter (Thomassen et al., 2023).

Besides adequate energy and nutrient availability, wild animals seek shelter from harsh weather and protection from predators and parasites, for example, insects. Observations of reintroduced ungulates have shown they use forest habitats for shelter (Cromsigt et al., 2018; Klich, 2017; Nicodemo & Porfírio‐da‐Silva, 2019; Pratt et al., 1986; Smagol et al., 2022; Zielke et al., 2019). Animals in fenced areas are restricted in their ability to mitigate temporal fluctuations in local resource availability by movement. This can make additional infrastructure, such as artificial weather shelter, watering points, and mineral licks, a necessity, but would be expected to have a limited effect on space‐use if natural alternatives are available (Van Laer et al., 2015). Animals also need to make a balanced judgment of landscape topography to minimize energy expenditure when moving across the landscape (Berti et al., 2022) while minimizing exposure to threat (Lundgren et al., 2022; Mas‐Carrió et al., 2024). This makes topography and vegetation density potentially important for space‐use preference (Berti, Rosenbaum, & Vollrath, 2025).

Herbivore space‐use also feeds back to impact the vegetation structure. A recent meta‐analysis showed that large herbivores decrease plant biomass and increase plot‐scale heterogeneity in a range of plant characteristics (Trepel et al., 2024). It remains unclear under which circumstances ungulates actively increase woody mortality in favor of herbaceous vegetation, but they can inhibit encroachment of already open patches on the landscape (Bühne et al., 2022; Kowalczyk et al., 2021; Nieszała et al., 2022; Tanentzap et al., 2023), occasionally maintaining high forage quality through the creation of grazing lawns (Hopcraft et al., 2010). This, in turn, increases landscape‐scale vegetation heterogeneity between patches, a correlate of landscape biodiversity (Estrada‐Carmona et al., 2022; Stein et al., 2014). Within patches of grassland, herbivores also increase the structural heterogeneity, with positive consequences for biodiversity (Bonavent et al., 2023; Dvorský et al., 2022; Gilhaus et al., 2013; Henning et al., 2017; Mata et al., 2025; Søndergaard, Ejrnæs, et al., 2025; Tanentzap et al., 2023). This aligns with the proposition that temperate European landscapes depend on large herbivores to keep parts of the landscape in open and semi‐open conditions in the absence of human management (Czyżewski & Svenning, 2025; Pearce et al., 2023, 2024; Svenning, 2002). Human land use has dominated vegetation structure for millennia in Europe (Bocherens, 2018; Søndergaard, Fløjgaard, et al., 2025), maintaining low densities of herbivores in areas dedicated to wood production (plantations, forests) alongside intensively grazed paddocks or agricultural fields, where woody growth is constantly suppressed. When management with sharp boundaries is discontinued and replaced by rewilding, the land use legacy, not least on vegetation openness, is a key regulator of ecosystem recovery trajectory. Without recurring disturbances, abandonment leads to widespread replacement of short vegetation and open woodlands by dense shrubs and trees. Such vegetation densification is consistent across plot (Bonavent et al., 2023; Tanentzap et al., 2023; Timmermann et al., 2015), landscape (Bühne et al., 2022; Tanentzap et al., 2023), and continental scales (Buitenwerf et al., 2018), with the diversity decline enhanced by climatic warming (Auffret & Svenning, 2022).

Considering all the evidence mentioned above, we expected to find similar diet‐driven space‐use predictors for cattle and horses, that is, preference for open habitats (hypothesis H1a) related to foraging (H1b), with potential diet‐driven divergence in space‐use during cold months with limited resources (H1c). Additionally, we expected horses and cattle to prefer open habitats and low topography when moving across the landscape to avoid locomotion resistance (H1d). Yet, we expected to find use of habitats with denser vegetation too, particularly for resting and shelter (H1e); at the same time, we expected limited attraction to artificial infrastructure (H1f) due to the availability of natural alternatives. We also expected animals to limit vegetation densification in intensively used open and semi‐open areas, but not in low‐use areas (H2a), while the vegetation in tree‐covered areas would not respond much to animal presence, as most of the live edible biomass is out of animal reach (H2b). This uneven animal impact on vegetation across the landscape is expected to increase vegetation heterogeneity at the landscape scale over time since animal introduction (H2c).

To test these hypotheses, we investigated the ability of year‐round grazing by reintroduced bovids and equids to reduce vegetation succession in a trophic rewilding area in Denmark. We tested the first set of hypotheses using telemetry data and a space‐use preference modeling framework to identify landscape drivers of animal space‐use. To test the second set of hypotheses, we investigated correlations of space‐use with changes in growing season vegetation density, as indicators of overall landscape productivity (mean) and habitat diversity (variance). The findings have general implications for trophic rewilding in Europe and other mild temperate climates where undisturbed vegetation succession typically results in densification and eventually closed‐canopy forest.

## MATERIALS AND METHODS

### Study site

The study site is a trophic rewilding experiment situated in an undulating moraine landscape in Eastern Jutland, Denmark (56.228 N; 10.575 E), which experienced a pronounced drought event in 2018, as did most of Europe. The vegetation is characteristic of dry heathland and dry acidic grassland developed on abandoned sandy fields in open areas and oak‐dominated broadleaf forests with occasional coniferous trees (*Pinus sylvestris*). Open, dry areas are in most places under encroachment with scattered shrubs and trees such as Scotch broom (*Cytisus scoparius*), pedunculate oak (*Quercus robur*), roses (*Rosa* ssp.), wild apple (*Malus sylvestris*), birches (*Betula* spp.), and blackthorn (*Prunus spinosa*). Part of the area has experienced periods of plantation forestry, of which the last were harvested in 2009, and shrubs were cut every 2–3 years to keep grasslands open. The management shift to trophic rewilding marked an intensification of the grazing regime in the closed‐canopy areas. In the grassland areas, rewilding marked a significant summer extensification and winter intensification of the grazing regime, due to the shift from seasonal grazing to year‐round grazing without supplementary feeding. The rewilding initiated in 2016 with an introduction of 12 mares (followed by one stallion in 2017) and 13 cattle (12 cows and 1 bull), after which the horse and cattle populations were allowed to fluctuate naturally. Animals were only removed if they were deemed unable to survive the winter, based on a conservative body condition system by which each individual was scored daily by the wildlife manager. The average density of animal biomass was ~120 kg ha^−1^ across the period, which fits well within the range of rewilding projects in Europe in areas with medium productivity (Fløjgaard et al., 2022), or only around 15%–20% of the growing season density in traditional intensive summer grazing in Denmark (Bonavent et al., 2023). During the winter 2019–2020, the populations were reduced to about 1/3 of the ~210 kg ha^−1^ peak in the autumn of 2019 (Appendix S1: Figure S2). In addition to the cattle and horses, the area hosts roe deer (*Capreolus capreolus*), European hare (*Lepus europaeus*), various rodent species, and transitory red deer (*Cervus elaphus*). Additional site information is provided in Appendix S1.

### Vegetation height and greenness

Vegetation height and density estimates for each pixel (10 × 10 m) were based on a one‐off national airborne Light Detection and Ranging (LiDAR) scan (ALS) of Denmark from 2015, that is, prior to project initiation, according to a recent model (Assmann et al., 2022). We constructed initial vegetation height classes to represent tree‐dominated pixels, where the majority of biomass was assumed unreachable for the animals (“tall” class), shrub‐dominated pixels, where most vegetation was assumed reachable for browsing, but likely also with substantial ground cover (“intermediate” class), and pixels dominated by short vegetation, assumed to be dominated by herbaceous vegetation with high forage quality (“short” class). Apart from vegetation height classes, we included the vegetation density from (Assmann et al., 2022) as a predictor of space‐use preference. Density was estimated as the ratio of ALS points returned by vegetation across all vertical height bins to the total number of returns in each cell (more in *Extended methods* in Appendix S1).

Previous studies have shown reasonable correlation between the normalized difference vegetation index (NDVI, a satellite‐derived product highly correlated with chlorophyll content, i.e., the “greenness” of a pixel) and biomass in open habitats, although the NDVI signal tends to saturate in canopy‐covered pixels (Borowik et al., 2013; Soubry & Guo, 2022). Hence, we use it as an estimate of biomass regrowth in semi‐open areas. We used Sentinel‐2‐derived growing season NDVI for two purposes: (1) to understand the spatial distribution of NDVI and how it correlates with animal preference and space‐use and (2) to quantify overall vegetation trends (mean) and heterogeneity (variance) at the scale of the rewilding area. For 2, we explored how the spatial resolution affected the calculated statistics by aggregating the pixels at different resolutions. Specifically, we calculated the mean NDVI values for spatial grids of 10 × 10 m in resolution up to 100 × 100 m, with increments of 10 × 10 m. We then obtained the mean and variance values at the total area level for all resolutions separately.

### Space‐use and space‐use preference modeling and prediction

GPS collars (VERTEX Plus, Vectronic Aerospace Gmbh, Berlin, Germany; time‐step: 30 min; average accuracy: 8–15 m) were deployed from December 2017 to March 2021, both included, spanning around three years of sampling. The number of GPS fixes (counts) in each pixel was used as a value for actual space‐use, which was transformed when used in regressions, and classified to represent low use (<4 fixes), intermediate use (>4 and <12 fixes), and high use (>12 fixes) in boxplots. To investigate what influenced space‐use preference of the animals within the rewilding area, we combined a state‐space model with a step‐selection function (SSF). Briefly, the state‐space model decomposed the movement process into different behavioral states and associated movement parameters. Hence, the number and type of mobility state categories used in some of the analyses are identified by this model, that is, three categories for cattle (resting, foraging, exploration), but only two for horses (resting/foraging and exploration). Then, the step‐selection model predicted the expected use of each pixel based on movement states and environmental factors and compared this with the actual use of each pixel. If the actual use was higher than predicted for a pixel, it got a preference score >0 (attraction), and if the actual use was lower than predicted for a pixel, it got a preference score <0. To predict the resulting space‐use preference, we selected a set of key variables: the category of vegetation height, the proximity to the shed (reciprocal of the distance in inverse meters), vegetation density (the density of vegetation‐derived LiDAR reflection points within a pixel), the landscape connectivity, and the vegetation greenness, that is, NDVI, a proxy for plant productivity. Vegetation height category was derived from ALS point clouds (see above). Landscape connectivity was calculated using the enerscape R package (Berti et al., 2022), using the energy costs as resistance matrices for movement. This is estimated based on locomotory theory of animals and topography, making connectivity higher across cells with lower energy expenditure for moving between them. NDVI values were aggregated into monthly NDVI images using the median values of the pixels to be used in preference modeling. Detailed information about our approach can be found in the Extended Methods in Appendix S1.

As we were interested in understanding both the overall habitat preference and how these varied seasonally and depending on the three mobility states identified for cattle (resting, foraging, and exploratory) and the two identified for horses (resting/foraging and exploratory), we ran three analyses per population. First, we performed the conditional logistic regression using the whole dataset (overall preferences). Then, we re‐ran the analysis, splitting the dataset into cold season (from November 1 to March 31) and warm season (April 1 to October 31) separately (seasonal preferences). Finally, we split the dataset based on the assigned movement states and re‐ran the analysis (state‐specific preferences). Spatial analyses were performed in the coordinate reference system ETRS89 UTM zone 32N (EPSG:25832; +proj = utm + zone = 32 + ellps = GRS80 + towgs84 = 0, 0, 0, 0, 0, 0, 0 + units = m + no_defs) at a resolution of 10 × 10 m.

### Statistical analyses of vegetation response to animal space‐use

To accommodate potential nonlinear relationships between initial vegetation height and animal space‐use correlated with vegetation greenness change, we used generalized additive models (GAM) to evaluate the relationships. We used the package *mgcv* and the thin plate regression splines in smoothing function *gam*. The initial, pre‐rewilding vegetation height and the GPS counts per species were included as smoothing terms to predict the NDVI change to find the best model fits. Then we tested whether models were improved by grouping the count data by mobility states. We compared models based on Akaike information criterion (AIC) and Bayesian information criterion (BIC). Concurvity between predictor variables was considered problematic if >0.5 (Kovács, 2022). Model summaries are available in Appendix S1: Table S3. Estimated marginal means (*emmeans* package) were used as an estimate of animal space‐use effects in the models. Categorical comparisons between vegetation height classes and space‐use classes were made using Kruskal–Wallis ANOVA followed by a pairwise post hoc comparison between groups with a Dunn test. Spearman's rank tests were used to test for significant correlations between two variables. The analyses and data wrangling were made with the packages *dplyr*, *purr*, *raster*, *viridis*, *tidyr* apart from the packages mentioned above, and visualizations were made with *ggstatsplot* and *ggplot2*, all in R 4.4.3 (R Core Team, 2025).

## RESULTS

### Animal space‐use

**FIGURE 1:**
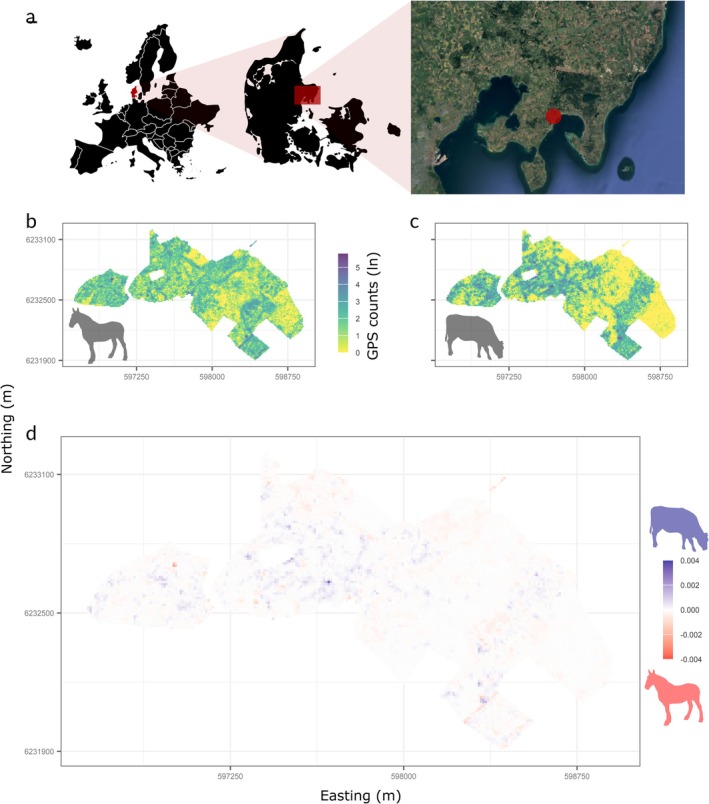
GPS fix counts for horses (b) and cattle (c) across the Rewilding Mols landscape (red dot on satellite image, panel a). Panel (d) shows the areas with relative dominance in space‐use by cattle (blue) and horses (red), and the gray areas with either no observations or no specific dominance. The following icons were used under CC BY‐3.0 Attribution License from the Noun Project: Europe (ilCactusBlu), Denmark (Sigit Mulyo Utomo). The following icons were used under CC0 1.0 Universal Public Domain Dedication from Phylopic: Cattle (*Bos primigenius taurus Linnaeus 1758*), Horse (*Equus ferus caballus Linnaeus 1758*). The satellite image was obtained from GoogleEarth.

**FIGURE 2:**
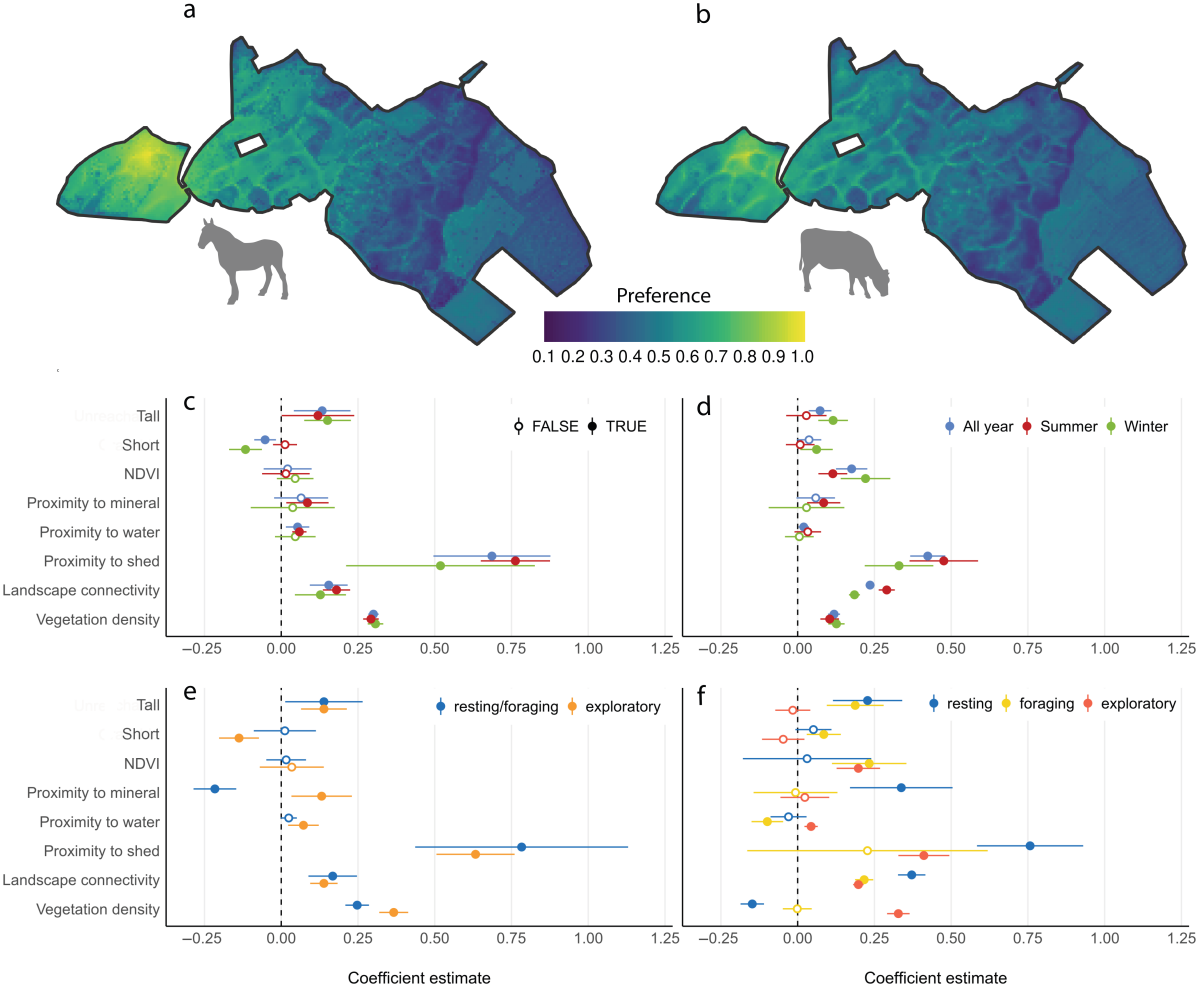
Preference maps for horses (a) and cattle (b) across the Rewilding Mols landscape. The coefficients (circles) and SEs (whiskers) for the modeling parameters predicting horse (c, e) and cattle (d, f) preferences resolved by season (c, d) and mobility states (e, f). The following icons were used under CC0 1.0 Universal Public Domain Dedication from Phylopic: Cattle (*Bos primigenius taurus Linnaeus 1758*), Horse (*Equus ferus caballus Linnaeus 1758*). NDVI, normalized difference vegetation index.

The actual space‐use of cattle and horses explained a limited proportion of the variation in one another (Spearman's ρ = 0.41, *p* < 0.001), suggesting a degree of space‐use complementarity (Figure 1; Appendix S1: Figure S6). Nonetheless, the space‐use preference of the two species, that is, the higher use of an area relative to model expectations, correlated strongly (Spearman's ρ = 0.87–0.95, Figure 2; Appendix S1: Figure S7), suggesting that strong attractors and/or repellers are similar. This partially supports the expected similarity in habitat preference (H1a). When the preferences were estimated per season (Figure 2c,d) and mobility states (Figure 2e,f), cattle exhibited more selective habitat use compared to horses, as indicated by larger differences between the seasonality and mobility subcategories for cattle than for horses. Cattle used a substantially smaller area during winter (mobility states calculated for 5233/11,491 pixels) than during summer (mobility states calculated for 7030/11,491 pixels, Appendix S1: Figure S8d,f), while horses used most of the area all year (mobility states calculated for 8769/11,491 and 7406/11,491 pixels during summer and winter, Appendix S1: Figure S8c,e). Particularly, cattle reduced the area where they spent time in the foraging state during cold winter months compared to the warm seasons. In contrast, horses used relatively more area being in the exploratory state during winter months. This supports the expected divergent strategies for coping with resource scarcity (H1c).

#### Vegetation characteristics

Vegetation height classes had limited ability to predict overall preference for any of the two herbivore species (Figure 2), including limited predictive power for short vegetation (<0.5 m), countering our expectation (H1a). Nonetheless, cattle had a preference for areas with short vegetation during winter, while horses seemed to avoid those areas during winters. Horses spent more time in areas with tall vegetation (maximum canopy heights >7 m) year‐round, while cattle only showed affinity for tall vegetation during winters. Both findings lean support to the expected habitat divergence during cold months with limited resources (H1c). Tall vegetation was also a positive predictor of horse preference in states corresponding to both foraging and exploration, but they did not seem to select for open areas when moving across the landscape, as expected if based purely on resistance avoidance (H1d). Cattle preferred areas with tall vegetation in resting and foraging states, but not when exploring, while short vegetation was a positive predictor in the foraging state only. Hence, our expected positive relationship between open areas with short vegetation and the mobility state corresponding with foraging (H1b) was confirmed for cattle but not for horses. Vegetation greenness (NDVI), which was highest in forests, was not important in predicting horse preferences, while it was a positive predictor for cattle preference, particularly during winter and when in exploratory and foraging states. LiDAR‐based vegetation density was a considerable positive predictor of preference for both species and strongest when in the exploratory state. Despite this nuance, the expected use of closed habitats was clearly demonstrated for both horses and cattle (H1e).

#### Internal landscape connectivity

Landscape connectivity inside the rewilding area, estimated using the energetic cost of moving across the terrain as a resistance matrix, was a significant space‐use predictor for both species as expected (H1d). Prediction power was highest for cattle, particularly during summer and when in resting mode, suggesting they rarely rest in steep areas.

#### Artificial infrastructure

Contrary to our expectation (H1f), the single‐most important explanatory variable for both species was the proximity to the only shed in the area, particularly for horses (Figure 2c,d), but with high variability (large error bars) for both species. The proximity to shed was a good predictor of horse preference across mobility states (Figure 2e), while for cattle the proximity to shed was a much stronger predictor when in the resting state than other movement states (Figure 2f). Other infrastructure (proximity to artificial water points and mineral licks) also had significant, but substantially smaller predictive power. Cattle showed attraction to mineral licks, particularly during summer and when in resting mode. Horses showed preference for water all year and mineral licks during summer, mainly in the exploratory state.

### Vegetation change and relationships with animal space‐use

#### Development in vegetation greenness

**FIGURE 3:**
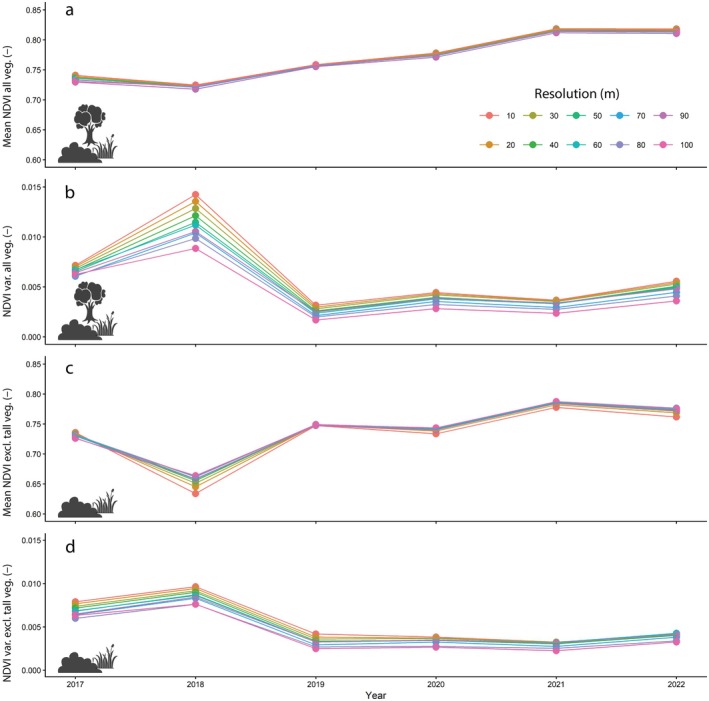
Mean (a, c) and variance (b, d) in vegetation greenness (normalized difference vegetation index [NDVI]) from 2017 to 2022 at the park level at resolutions ranging from 10 to 100 m, including all pixels (a, b) and without pixels in the unreachable vegetation class representing mainly forests (c, d). The following icons were used under CC BY‐3.0 Attribution License from the Noun Project: Grass (Nanik haq), Shrub (wahab marhaban), Tree (kareemov).

We found a weak increase in mean NDVI from 2017 to 2022 when all pixels are considered (Figure 3a). When the pixels with tall vegetation (trees) were excluded, there was an obvious drop in mean NDVI in 2018, when the major drought occurred, followed by a quick recovery in the following years of 2019 and 2020 (Figure 3c).

Contrary to our expectation that vegetation heterogeneity should increase with time since conversion to rewilding management (H2c), NDVI heterogeneity remained lower after the 2018 drought than before, irrespective of whether pixels with tall vegetation were included. The increase in NDVI heterogeneity in 2018 was much more pronounced when trees were excluded (Figure 3b compared to Figure 3d). This suggests that while trees drive most of the temporal variation in mean NDVI (i.e., due to higher NDVI values), the temporal variation in NDVI heterogeneity is mainly governed by the more dynamic shorter vegetation classes. The effect of spatial resolution (the distance within which pixels were aggregated to calculate mean and variance) was relatively small, suggesting limited patchiness, that is, there are pixels with high and low NDVI spread across the study area.

**FIGURE 4:**
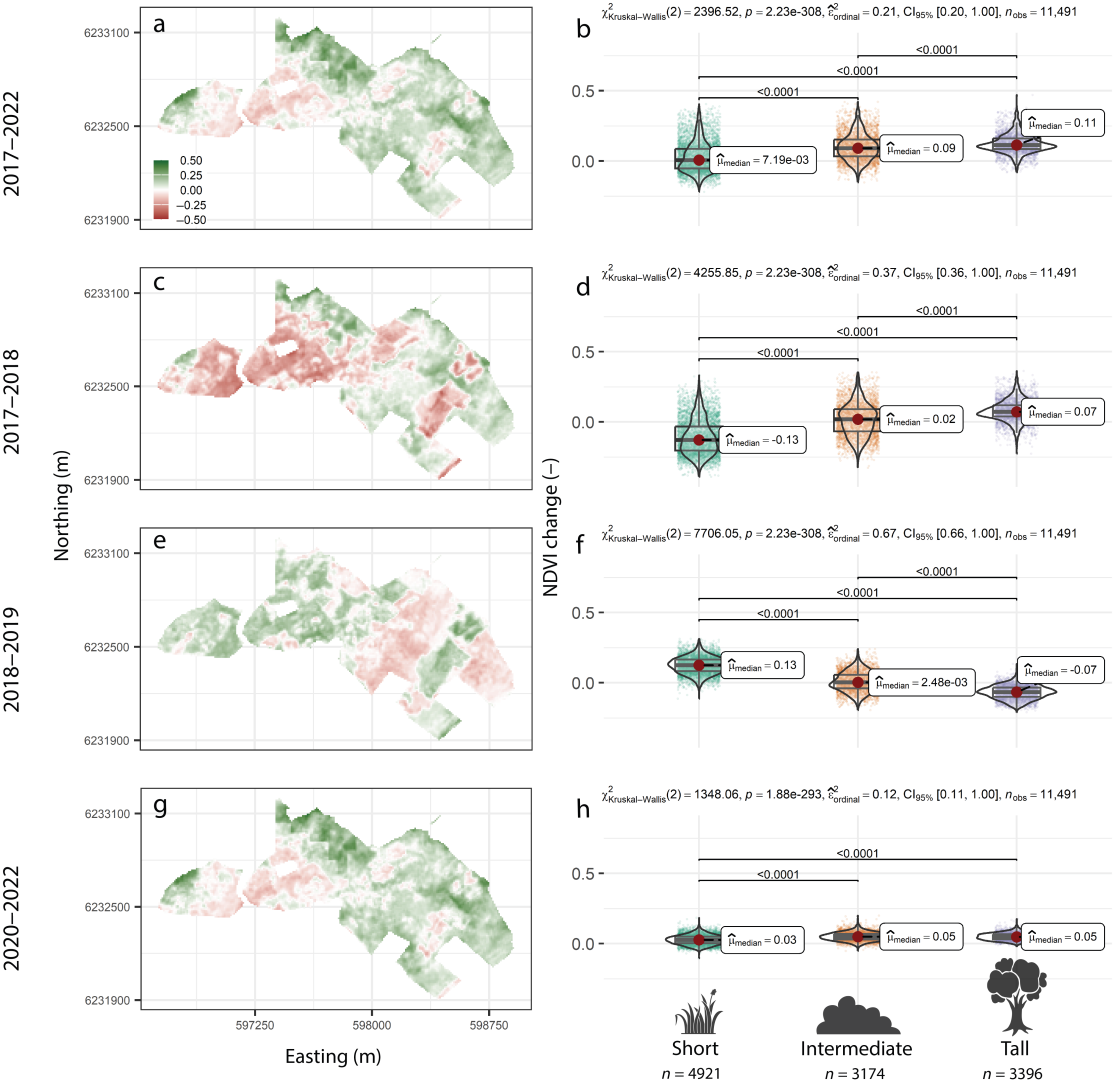
Normalized difference vegetation index (NDVI) change across all vegetation from 2017 to 2022 (a, b), 2017–2018 (c, d), 2018–2019 (e, f), and 2020–2022 (g, h) shown on maps (a, c, e, g), and in boxplots for the three vegetation classes (b, d, f, h). In the boxplots, the horizontal line and red dot show the median, the box edges show quartiles, and the violin plot shows the kernel density distributions of the data. *p*‐values above the plot show the results of Kruskal–Wallis tests, and the *p*‐values for the pairwise comparisons (Dunn tests) are shown when significant. The following icons were used under CC BY‐3.0 Attribution License from the Noun Project: Grass (Nanik haq), Shrub (wahab marhaban), Tree (kareemov).

During drought response (2017–2018) and recovery (2018–2019), the three vegetation classes responded differently (Figure 4b,d,f). While short vegetation showed a mean decrease of −0.12 NDVI units, trees increased by 0.07 NDVI units on average during the drought. Similarly, short vegetation showed enough NDVI increase 1‐year post‐drought to bring the average NDVI back to pre‐drought levels. In contrast, trees kept increasing by a similar amount as during the drought. When the drought response was plotted against the drought recovery (Appendix S1: Figure S9a), the correlation decreased in the order of all pixels (Spearman's ρ = −0.707) > short (ρ = −0.662) > intermediate (ρ = −0.539) ≫ tall vegetation (ρ = 0.103) (all *p* < 0.001). This shows that while trees show low sensitivity and high resistance to drought, grasses and shrubs are generally sensitive but resilient, that is, recovered relatively fast after disturbance. Importantly, all three vegetation classes had pixels showing both positive and negative NDVI changes during both time windows, which motivates the analysis of how much of this variation space‐use can explain within each class.

#### Vegetation response to animal space‐use

Our results support that herbivores exert some control on regrowth in open and semi‐open areas, but little in areas dominated by tall vegetation, as expected (H2a,b). For all time periods, adding the space‐use conditioned by vegetation height classes improved the predictive power of space‐use markedly relative to when added unconditioned (increased explained deviance from 1%–29% to 10%–67%). This clear interaction between vegetation height class and space‐use effects on NDVI change suggests that the impact of herbivores is not the same in magnitude and/or direction in all vegetation types, confirming the importance of the initial vegetation structure, largely a legacy of previous land use history, to understand vegetation responses to animals. The estimated marginal means (Appendix S1: Figure S10) for the models with space‐use conditioned by vegetation classes showed a highly significant positive effect of both horse and cattle space‐use on NDVI‐change across the entire period (2017–2022), during drought recovery (2018–2019) and following the severe population reduction (2020–2022), and a negative effect during the drought (2017–2018). This confirms the importance of considering multiple stressors and stress history to interpret animal effects on vegetation trajectories.

**FIGURE 5:**
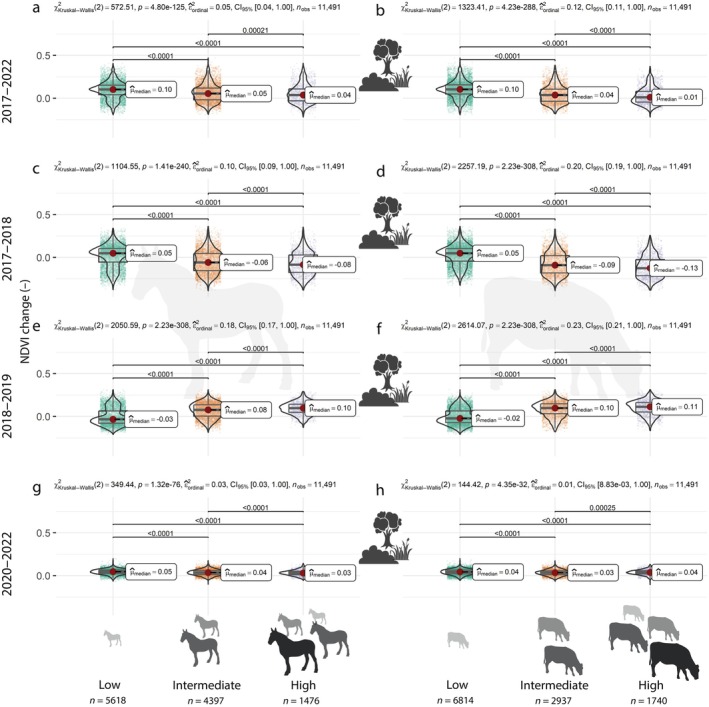
Normalized difference vegetation index (NDVI) change across all vegetation from 2017 to 2022 (a, b), 2017–2018 (c, d), 2018–2019 (e, f), and 2020–2022 (g, h) for horses (a, c, e, g), and cattle space‐use (b, d, f, h). In the boxplots, the horizontal line and red dot shows the median, the box edges show quartiles, and the violin plot shows the kernel density distributions of the data. *p*‐values above the plot show the results of Kruskal–Wallis tests, and the *p*‐values for the pairwise comparisons (Dunn tests) are shown when significant. The following icons were used under CC BY‐3.0 Attribution License from the Noun Project: Grass (Nanik haq), Shrub (wahab marhaban), Tree (kareemov). The following icons were used under CC0 1.0 Universal Public Domain Dedication from Phylopic: Cattle (*Bos primigenius taurus Linnaeus 1758*), Horse (*Equus ferus caballus Linnaeus 1758*).

We found a negative correlation between NDVI change and space‐use of both horses and cattle across the entire period 2017–2022 (Figure 5a,b; Appendix S1: Figures S11 and S12). Looking at each vegetation class separately for the same period, vegetation in the intermediate height class (shrubs) clearly had the most positive NDVI change when animals used an area very little, while the tall vegetation class showed nonsignificant differences between space‐use intensity classes (Appendix S1: Figure S12). In the low vegetation class corresponding to open grasslands, there was a weak increase in NDVI with increasing use by horses, but an NDVI decrease with increasing space‐use intensity by cattle (Appendix S1: Figures S11a,b and S12a,b). The higher ability of cattle space‐use to explain NDVI change was confirmed by the GAM model fits. When cattle space‐use was added together with initial vegetation height, the model explained 22% of the deviance in NDVI change across the entire study period (2017–2022), while adding horse space‐use to predict NDVI change did not improve the model beyond the model with only initial vegetation height as a predictor (*R* ^2^_adj_ = 0.19, Appendix S1: Table S3).

Effects of animals on NDVI change were also highly significant during the drought and in the following recovery, but also here adding cattle space‐use improved model fits relative to adding horse space‐use, although the estimated marginal means were similar between the two species (Appendix S1: Figure S10). Across all vegetation classes, a marked negative correlation between NDVI change and space‐use during the drought (Appendix S1: Figures S10a,b and S11c,d) was compensated by a similar‐sized positive relationship during recovery (Appendix S1: Figures S10a,b and S11e,f). In fact, the estimated marginal means suggest the positive effect of animals on NDVI after the drought is slightly higher for both species than the negative effect they had during the drought (Appendix S1: Figure S10). To further support this relationship, we again looked at the trends within each vegetation height class. In the tall vegetation class, the difference between space‐use classes was small, but pixels with higher animal use showed a higher NDVI increase, particularly in post‐drought recovery (Appendix S1: Figures S13e,f and S14e,f). NDVI for the intermediate vegetation height class showed a stronger reduction and recovery when animal space‐use increased (Appendix S1: Figures S13c,d and S14c,d). For low vegetation, the impact of horses and cattle was different. Areas with intensive horse use showed a smaller decrease in NDVI compared to areas of lower use, while there were only small differences between space‐use classes in the drought recovery phase (Appendix S1: Figures S13a and S14a). For cattle, there was a clear negative relationship between space‐use and NDVI change in the low vegetation class during the drought and a clear positive relationship in the recovery phase (Appendix S1: Figures S13b and S14b). Only cattle count improved the GAM model fits beyond what was explained by the initial vegetation height only (Appendix S1: Table S3), despite both species having similar estimated marginal mean effects (Appendix S1: Figure S10). Models with initial vegetation height and cattle space‐use explained 43% of the deviance in NDVI change during droughts compared to 39% for a model with only initial vegetation height and a model with horse space‐use. For NDVI change during drought recovery, the explained deviance was 72% for a model with cattle space‐use compared to 70% for a model with only initial vegetation height and 71% for one with initial vegetation height and horse space‐use (Appendix S1: Table S3). Hence, adding cattle space‐use to the models explains an additional 4% and 2% of the deviance in landscape‐scale NDVI change during and one year after a severe drought.

Space‐use did not explain much apparent variation in NDVI change following the drop in grazing pressure to ~1/3 in early 2020 (2020–2022) (Figure 5; Appendix S1: Figure S15), reflected in the poor ability of the GAM models to predict this change (16% explained deviance in best model, Appendix S1: Table S3). Nonetheless, the estimated marginal means were positive for both cattle and horse space‐use when grouped by vegetation height classes (Appendix S1: Figure S10). This suggests that within each vegetation class, the NDVI increased more where the animal use had been highest on average across the GPS‐deployment period, that is, with the most pronounced release from top‐down grazing control. Yet, the poor signal in the boxplots and GAM models suggest that it is not a very strong pattern. Further, the NDVI change following the population reduction did not correlate well with the variation in the NDVI change during (Spearman's ρ = <0.01–0.16) or following (Spearman's ρ = <0.01–0.10) the drought. Hence, the areas showing the highest increases in greenness after grazing pressure was reduced were different from the ones showing the largest responses to drought.

Conditioning the animal space‐use by mobility states did not improve the overall ability of the models to predict NDVI change (Appendix S1: Table S3). The estimated marginal means of space‐use conditioned by mobility states did not vary between states for cattle, suggesting that the effect of cattle presence on NDVI change was the same irrespective of the identified mobility states (Appendix S1: Figure S10). In contrast, the estimated means for horses were different between resting/foraging and exploratory states, although the differences were relatively minor. We note that the number of observations in the models including mobility states was reduced compared to the full model in a non‐random way. This is a result of mobility states only being calculated for pixels where enough GPS fixes were available to calculate turning angles and movement speed (*n* = 5233–7988 for cattle and *n* = 7401–9850 for horses relative to *n* = 11,491 for full model). Despite this potential caveat, it seems robust that including the GPS‐inferred mobility states did not improve the explanatory power of NDVI change across any of the tested time windows (Appendix S1: Table S3).

## DISCUSSION

Space‐use preferences for cattle and horses were similar and shaped by both vegetation characteristics (max height, greenness, and density) and landscape connectivity (locomotive resistance), and mostly supporting our expectations (H1). Attraction to open spaces was significant, although less clear than expected, and we confirmed the expected habitat divergence during periods of resource scarcity. Avoiding locomotive resistance also guided preference, particularly for cattle, while we found an unexpected large attraction to an artificial shelter in the area, which was strongest for horses. Despite confirming that animal space‐use reduced vegetation densification in intensively used open areas, this did not lead to the expected increase in the overall landscape‐scale vegetation heterogeneity since animal introduction (H2). This was potentially due to strong responses to drought blurring animal impacts. More detailed discussions are found below.

### Animal space‐use

**FIGURE 6:**
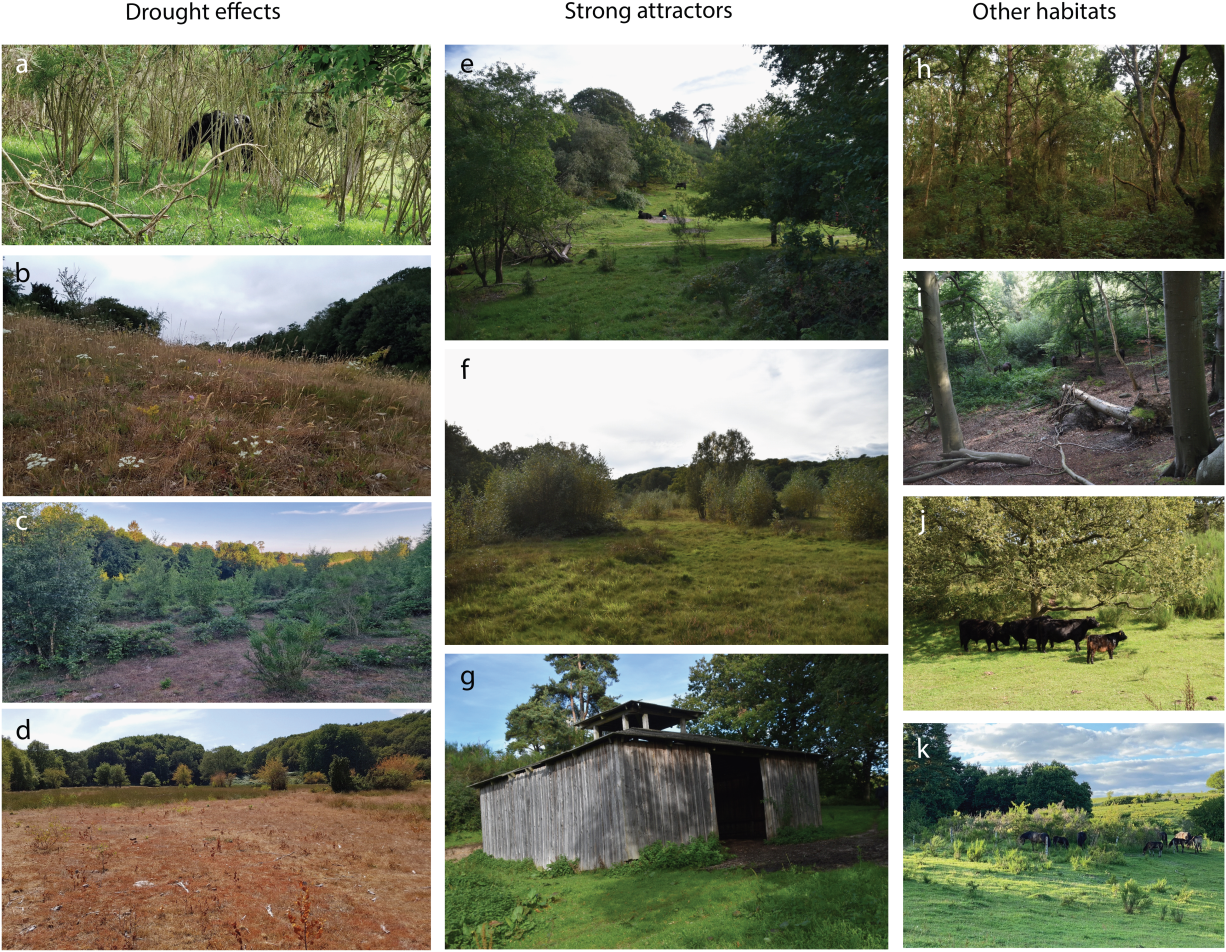
(a) Exmoor horses grazing the herbaceous ground cover under the semi‐open scotch broom canopy (*Cytisus scoparius*) during the drought of 2018. (b) Forbs favored over graminoids during the 2018 drought. The herbaceous layer was clearly more severely hit by the drought compared to shrubs and trees (c), with only the wetter parts of the landscape maintaining ground vegetation (d) unless shaded by shrubs (a). (e) Cattle resting close to an artificial water point in a frequently used landscape depression. (f) The shed, which was a very strong attractor for particularly horses. (g) The popular lowlying meadow area on a cloudy summer day. (h) A mixed forest in a low‐use area with dense bramble in the understory. (i) Horses foraging in a semi‐open forest. (j) Cattle enjoying the shade under a tree in a heavily grazed park landscape typical for the area. (k) Typical dry grassland in a highly used area. *C. scoparius* encroachment accelerated after the drought, but the exclosures show a clear ability of animals to moderate it. Photos were taken by: Morten D. D. Hansen (panel a–d), Jens‐Christian Svenning (panel e–j), Frederik N. Philipsen (panel k, with permission).

Cattle showed higher seasonal variation in space‐use and higher preference for energetically efficient mobility than horses. The generally lower predictability of horse preference relative to cattle could be an effect of continuous year‐round use of most of the rewilding area by horses. In contrast, cattle reduced their energy use by limiting their foraging activities to very specific grassland patches during winter. Indeed, seasonal eDNA analyses of dung from the animals at the studied site showed that diet overlap decreased during winter (Thomassen et al., 2023). Horses supplemented graminoids in their winter forage with mainly tree biomass, while cattle preferred thorny shrubs (mainly *Rubus*, which is evergreen in the area) for supplementing their grass and forb‐based diet. This scarcity‐driven diet divergence aligns with previous findings from temperate Europe (Cromsigt et al., 2018; Pratt et al., 1986; Putman et al., 1987) and elsewhere (Janis, 1976; Scasta et al., 2016), as well as with the seasonal habitat divergence by bovids and equids in a nearby rewilding site (Rech et al., 2025). Vegetation greenness (NDVI) correlated with cattle preference year‐round, but particularly during winters, when they feed on evergreen shrubs, confirming bovid attraction to greenness found in a comparable rewilding site (Rech et al., 2025). During droughts both species have been observed to feed extensively on herbaceous vegetation under the *C. scoparius* (Figure 6), which could add to the positive correlation between NDVI and space‐use. Yet, preferential grazing under semi‐open canopies is not necessarily limited to extreme events, in line with observations from the study area and with other European studies (Klich et al., 2023). Similarly, we found that animals preferred areas with high vegetation density, which is highest under shrubs and young trees (see *Extended methods* in Appendix S1). This is particularly pronounced when moving across the landscape. Hence, we speculate whether the semi‐open shrub‐forests may in fact offer a favorable balance between protection, forage availability, and shelter on one side and friction on the other. This seems plausible given the relatively open *C. scoparius* scrub/forest, the obvious difference in shrub density inside and outside exclosures under their canopies, and abundant trails suggesting frequent animal use in the area. No matter what, and as others have shown before, our results support that both horses and cattle use closed‐canopy habitat when available (Klich et al., 2023; Popp & Scheibe, 2014; Pratt et al., 1986).

Topography is a strong constituent of the landscape connectivity variable (Berti et al., 2022). This probably explains why landscape connectivity was a better space‐use predictor for the relatively heavier cattle than for horses, despite both species generally selecting for lower energetic expenditure when moving across the landscape. Consequently, topography is an important co‐determinant of the distribution of animal effects on the landscape, leaning additional support to the claim that geodiversity should be weighted highly during protected area prioritization (Bergin et al., 2024). This is particularly true in lowland regions like Denmark, where topography is generally an important determinant of plant diversity at the 1–100‐m scale (Moeslund, Arge, Bøcher, Dalgaard, Odgaard, et al., 2013; Moeslund, Arge, Bøcher, Dalgaard, & Svenning, 2013).

Finally, we found that both species were strongly attracted to the human‐built infrastructure, notably the shed (Figure 6). This was unexpected as cattle have been found to prefer natural shelter over artificial when available (Van Laer et al., 2015), as in the study area. Yet, the observed use of abandoned houses by horses in the Chernobyl exclusion zone (Gashchak & Paskevych, 2019) indicates that animals sometimes prefer human‐built structures for shelter, even when natural shelter is abundant. A similar attraction to human‐built resources was recently found in a nearby rewilding site (Rech et al., 2025), but in an area with less natural shelter. We speculate that the attraction to artificial water points could also be reduced if better access to the natural water resources in the area had been secured. Flooding concerns for downstream holiday homes have caused large stretches of the local creek to be fenced off, while road regulation has cut off access to the water resources along the shore. The centennial survival of feral horses on the Sable Island sand bar in Canada without access to artificial shelter, closed habitats, nor artificial water points (Government of Canada, n.d.) is a testament to the ability of large animals to find the resources they need even in extremely weather‐exposed habitats with limited fresh‐water resources.

### Vegetation structure and landscape‐scale diversity

Animals space‐use added significant predictive power to the models explaining vegetation structure, especially within vegetation classes, and more for cattle than horses. As expected, the impact of herbivores was strongest in frequently used areas, with highest sensitivity in grassland vegetation (i.e., short and intermediate height vegetation classes). The structuring of semi‐open grassland vegetation by animal use is in line with previous observations from temperate Europe, where year‐round grazing by feral cattle and horses has been found to restrict shrub encroachment (Ejrnæs et al., 2024; Köhler et al., 2016) and moderate the relative abundance of long grass, short grass, and bare ground in grasslands similar to the study area (Gilhaus et al., 2013; Henning et al., 2017). Woody growth inhibition is a common result of winter browsing (Kullberg & Bergström, 2001) promoting semi‐open vegetation rather than uniform succession toward homogeneous canopies. In areas like Northern Europe, with much less woody cover than historical baselines, some initial adjustments of the vegetation should be expected during rewilding. For example, woody regrowth after terminated agricultural management added significant amounts of aboveground biomass to the landscape in the “Southern Block” at Knepp (UK) during the first decade of rewilding (abandonment) until partly controlled by the introduction of large mammals (Tanentzap et al., 2023). Such shrubification was not seen in the middle and northern blocks, where large grazers were introduced to former agricultural land from the beginning of the project (Bühne et al., 2022). The missing reversal of the initial shrubification after animal introductions, but maintenance of open patches in the Southern Block, is in line with the formation of quasi‐stable mosaics of vegetation structure by herbivores (Olff et al., 1999; Pausas & Bond, 2022; Vera et al., 2006). Higher structural heterogeneity in vegetation is indeed found to correlate positively with large herbivore densities at the global scale (Wang et al., 2023), and palaeo‐evidence suggests that semi‐open landscapes with light woodland‐grassland mosaics is a reasonable baseline for herbivore‐rich European landscapes before human domination (Czyżewski & Svenning, 2025; Davoli et al., 2024; Pearce et al., 2023, 2024; Sandom et al., 2014). Based on this, our naïve expectation was that the uneven herbivore pressure on the vegetation across the landscape would increase the heterogeneity of the vegetation structure at the landscape scale over the course of the rewilding period. In contrast, the landscape‐scale heterogeneity gradually decreased over time, except for the intense drought year of 2018. The drop in heterogeneity after the drought relative to pre‐drought heterogeneity was mirrored by a post‐drought increase in mean NDVI. Consistent with anecdotal observations from the field, we identify two potential mechanisms for this, which may act in concert, most importantly, acceleration of the ongoing shrub encroachment in the semi‐open areas, particularly by *C. scoparius* (Figure 6), as a response to the significant drop in grazing pressure. Increased woody cover in the previously open landscapes would make the vegetation characteristics of the semi‐open areas approach the tree‐covered areas. Consequently, the spatial heterogeneity in NDVI at the landscape scale would be reduced while the mean NDVI would increase. A smaller contribution could come from drought‐accelerated transitions from graminoid to forb cover (Figure 6), maintained in a more forb‐rich state by intensive post‐drought grazing pressure, as large grazers tend to favor forbs over graminoids (Bråthen et al., 2021; Eskelinen et al., 2022; Nelson et al., 2025). A grass‐to‐forb transition could contribute to the lower satellite‐based temporal heterogeneity, due to the less pronounced biomass phenology of forbs relative to graminoids.

Animal space‐use consistently explained additional deviance in NDVI change during and after the drought relative to models without animal use, particularly in the intermediate and short vegetation height classes. Despite their drought sensitivity, herbs and shrubs also showed high ability to quickly regain their pre‐drought NDVI, that is, high resilience, particularly under intermediate animal pressure. The positive yet weaker correlation between average animal space‐use and NDVI change within each vegetation class after the severe population reduction suggested that certain areas within each vegetation class increased in greenness as a response to grazing pressure reduction but were more evenly distributed across the vegetation classes. Combined with the poor correlation between the pixels that responded to grazing reduction and the ones that responded to drought, we interpret this as productivity being mainly top‐down controlled by herbivores in some areas and bottom‐up controlled by abiotic conditions in others. Overall, this shows how environment, extreme events and herbivory interact to shape overall vegetation responses, but further studies of when top‐down and bottom‐up control is most important are warranted. Understanding their interactions will become increasingly important to understand vegetation change in a future with more frequent extreme events, notably droughts (Liu et al., 2025; Zhang et al., 2025) driving increased fire risk in temperate Europe (El Garroussi et al., 2024).

### Limitations of the study

Our findings prompt thorough consideration of the infrastructural design of rewilding projects to minimize interference with natural behavior. Yet, we note that there was only one shelter and one mineral lick in the study area, making conclusions based on their location sensitive to confounding attractors. Nonetheless, our results point to the need for a better understanding of the effects of infrastructure on animal space‐use.

Furthermore, our results showed that animal space‐use adds 2%–4% predictability of NDVI changes compared to models with vegetation height only. This may seem like small model improvements but given the many variables adding noise to potentially obscure the relationship (interannual differences in weather, variation in animal density, the limited number of collars, the fact that NDVI is only a proxy of vegetation properties measured at 10 × 10 m resolution, etc.), we believe it reflects a solid and important signal, similar in range to other remote‐sensing studies of animal effects on vegetation (e.g., Wang et al., 2023). An important caveat of passive spectral reflectance methods, such as NDVI, is that they mainly yield information on the uppermost vegetation layer. In (semi‐)open landscapes, NDVI usually correlates reasonably well with vegetation biomass (Soubry & Guo, 2022), but we would miss increased layering of the vegetation under canopies, which could weaken our relationships between NDVI and animal space‐use. Using active sensing (i.e., ALS), a recent study of European bison space‐use preference in the Bialowieza Forest in Poland showed that understory development (a similar variable as our vegetation density) was a better predictor of bison preference than canopy cover per se (Klich et al., 2023). Repeated ALS campaigns could be used to explore this.

Finally, we note that the telemetry data were based on four collars, two on each species, and hence, as any other telemetry study, the findings rely on the collar‐bearing animals to behave representatively of the group they represent, that is, their species in this case. As the animals are visually examined daily, and the wildlife managers have never noted any abnormal behavior by the collared animals specifically, we have no reason to believe they behaved differently than the rest of their group. Comparison with observational datasets could improve this, and it could improve our interpretations of the mobility mode, which did not add predictive power to our models in this study.

## CONCLUSIONS

Our study demonstrates that reintroduction of large grazers can limit vegetation densification in highly used areas. We also found that the vegetation in highly used areas was more sensitive yet resilient to drought. In contrast, the increase in vegetation greenness that followed a substantial reduction in herbivore population size showed an unclear relationship with animal space‐use. Neither did we see an increase in overall vegetation heterogeneity at the landscape scale, which we expected to follow logically from the ability of animals to keep highly used patches open, while shrubs would encroach open patches with low use. Our findings regarding animal space‐use lent partial support to our main expectations; the overall drivers were similar for the two species (e.g., locomotion resistance, vegetation characteristics), although cattle were generally more selective than horses. We also found support for the expected divergence of habitat use during cold months, where resource availability is lowest. Consequently, the functional diversity represented by two species of herbivores, which are often perceived as functionally similar large grazers in Northern European nature management contexts, can likely amplify the positive effects on vegetation heterogeneity and plant diversity beyond a one‐species scenario. The most surprising result was the strong attraction to the artificial infrastructure, notably the shed, warranting careful consideration of use and placement of artificial infrastructure in rewilding areas. Similarly, the attraction to artificial water points could perhaps be avoided if the fencing allowed better access to the natural water resources available in the area. Considering how animal use shaped the drought responses of vegetation, grazers will likely become increasingly important for light‐demanding species in a future where climate warming and extreme weather events are expected to accelerate vegetation densification in mild temperate Europe, particularly if compared with alternative nature management techniques, for example, land abandonment (passive rewilding). In summary, our work adds to the growing body of evidence that trophic rewilding with the reintroduction of large grazers in natural densities offers an important strategy to maintain structurally diverse landscapes via autonomous ecological processes in temperate Europe. This has the potential to make ecosystems more robust to the ongoing and predicted future pressures from various global change factors, such as climatic warming, extreme events, and alien plant invasions, all contributing to increased ecosystem novelty and uncertainty (Svenning, Buitenwerf, & le Roux, 2024).

## AUTHOR CONTRIBUTIONS

Jeppe Å. Kristensen, Robert Buitenwerf, Emilio Berti, and Jens‐Christian Svenning conceived the paper. Jeppe Å. Kristensen, Robert Buitenwerf, Emilio Berti, and Jens‐Christian Svenning designed and conducted the analyses. Jeppe Å. Kristensen led the writing, with contributions throughout the process by Robert Buitenwerf, Emilio Berti, and Jens‐Christian Svenning. Oskar L. P. Hansen, Simon D. Schowanek, Morten D. D. Hansen, Rasmus Ejrnæs, Kent Olsen, and Signe Normand gave valuable inputs to the manuscript. All authors approved the final submitted version.

## CONFLICT OF INTEREST STATEMENT

The authors declare no conflicts of interest.

## Supporting information


Appendix S1.

## Data Availability

Data and code are available in Zenodo as follows: space‐use modeling (Berti, Kristensen, & Svenning, 2025), https://doi.org/10.5281/zenodo.17376061; vegetation responses to space‐use (Kristensen, 2025), https://doi.org/10.5281/zenodo.17896843.
