# Supplementary material for: Space‐use by feral cattle and horses shapes vegetation structure in a trophic rewilding area
Source: Ecol Appl. 2026 Feb 4;36(1):e70170. doi: 10.1002/eap.70170 (PMC12871458; doi:10.1002/eap.70170)
Supplement: Supplementary file 1 — Appendix S1. [file EAP-36-e70170-s001.pdf]

## Appendix S1

# Space-use by feral cattle and horses shapes vegetation structure in a trophic rewilding area

Jeppe Å. Kristensen, Robert Buitenwerf, Emilio Berti, Oskar L. P. Hansen, Simon D. Schowanek, Rasmus Ejrnæs, Morten D. D. Hansen, Kent Olsen, Signe Normand, Jens-Christian Svenning

*Ecological Applications*

Extended methods ........ 2
- Study site ........ 2
- Animal populations ........ 3
- ALS-based vegetation height and density ........ 5
- Vegetation greenness measured as NDVI ........ 5
- GPS collar data for movement analyses ........ 5
- State-space modelling ........ 6
- Step-selection function ........ 8

References ........ 21

Display items

Figure S1-S15

Table S1-S3

# Extended methods

## *Study site*

Rewilding Mols is a trophic rewilding experiment owned and run by the Natural History Museum in Aarhus. It is situated in an undulating moraine landscape in Eastern Jutland (56.228N; 10.575E). An old coastline from the Mesolithic transgression (~6-8,000 BP) divides the landscape into a flat low-lying uplifted seabed (elevation 1-2 m above sea level), and the upper moraine landscape (35-55 m a. s. l., Figure S11a). A tree-covered slope lies in between (Figure S11b,c). The elevated areas are dominated by loamy sand deposited in terminal moraines (formed ~18-19,000 BP, (Houmark-Nielsen et al. 2006). The lower-lying areas towards the coast (SE) are dominated by marine sand from the Mesolithic transgression, but with substantial organic deposits due to groundwater saturation in landscape depressions. The mean annual temperature for the municipality (Syddjurs) was 8.9°C and the mean annual precipitation was 678 mm $year^{-1}$ for the period 2006-2015 (trap.lex.dk). The area experienced a pronounced drought event in 2018, as did most of Europe.

The vegetation is characteristic of dry heathland and dry acidic grassland developed on abandoned sandy fields in open areas and oak dominated broadleaf forests with occasional coniferous trees (*Pinus sylvestris*). On the Mesolithic seabed, the open ground is covered with a mixture of dry, acidic grassland, acidic meadow and alkaline fen interspersed with alder-ash swamp forest. The sloping area along the former coastline is covered with broad-leaved forest, dominated by oak (*Quercus robur*) and beech *(Fagus sylvatica)*. In the more elevated parts of the moraine landscape open, dry areas are in most places under encroachment with scattered shrubs and trees such as Scotch broom (*Cytisus scoparius*), pedunculate oak (*Quercus robur*), roses (*Rosa* ssp.), wild apple (*Malus sylvestris*), birches (*Betula* spp.), and blackthorn *(Prunus spinosa)*.

Typical for areas of marginal agricultural value in Denmark, part of the area has experienced periods of plantation forestry, of which the last were harvested in 2009. Most of the broad-leaved forests have regrown since a historical low in tree cover just after World War II, with the oldest oak and beech trees on the steeper slopes dating back to the early 1900s. There is no record of intensive forestry in the lower-lying areas to the east, but the presence of drainage ditches suggests there has historically been some degree of silvi- and/or agriculture. The open areas within the fenced rewilding area were also grazed with cattle prior to 2016, when the rewilding project initiated, yet in a traditional summer-grazing scheme, with higher densities of animals in smaller paddocks. To keep grasslands open, areas were occasionally cut (every 2-3 y) with machines to restrict shrub encroachment, with the removal of *Cytisus scoparius* being of particular focus. Hence, the management shift to trophic rewilding marked an intensification of the grazing regime in the closed-canopy areas. In the grassland areas, it marked a significant summer extensification and winter intensification of the grazing regime, due to the shift from seasonal grazing to year-round grazing without supplementary feeding.

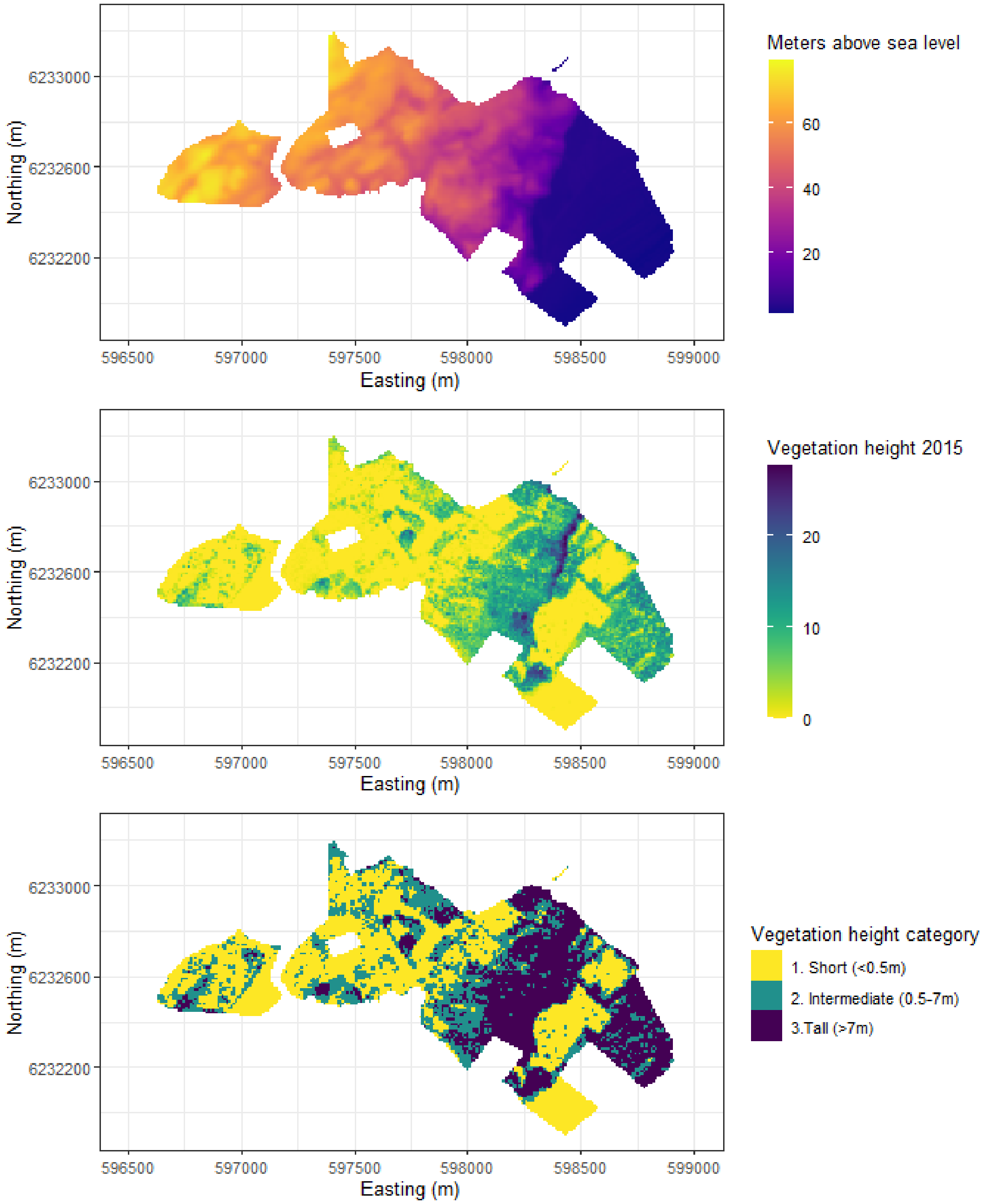


***Figure S1****: Digital elevation model (a) and initial vegetation height model (b) and corresponding vegetation classes (c) for the study area. In a, the delineation between the lowlying, flat area (purple) and the upper moraine landscape (orange-yellow) is clear.*

## *Animal populations*

In 2016, 12 mares and 13 cattle (12 cows, 1 bull) were released into the area. In the summer of 2017, a stallion was added. The horse and cattle populations were allowed to fluctuate naturally. Animals were only removed if they were deemed unable to survive the winter, based on a conservative body condition system by which each individual was scored daily by the wildlife manager. Hence, the herbivore density fluctuated over the experimental period from ~70 kg $ha^{-1}$ just after introduction to a peak at ~210 kg $ha^{-1}$ (35 horses and 41 cattle) in the autumn of 2019, before the populations dropped

back to ~70 kg ha$^{-1}$ (21 horses and 15 cattle, Figure S1) in the early spring of 2020. The average density was ~120 kg ha$^{-1}$ across the period, which fits well within the range of rewilding projects in Europe in areas with medium productivity (Fløjgaard et al. 2022), or only around 15-20% of the growing season density in traditional intensive summer grazing in Denmark (Bonavent et al. 2023). In addition to the cattle and horses, the area hosts roe deer (*Capreolus capreolus*), European hare (*Lepus europaeus*), various rodent species, and transitory red deer (*Cervus elaphus*).

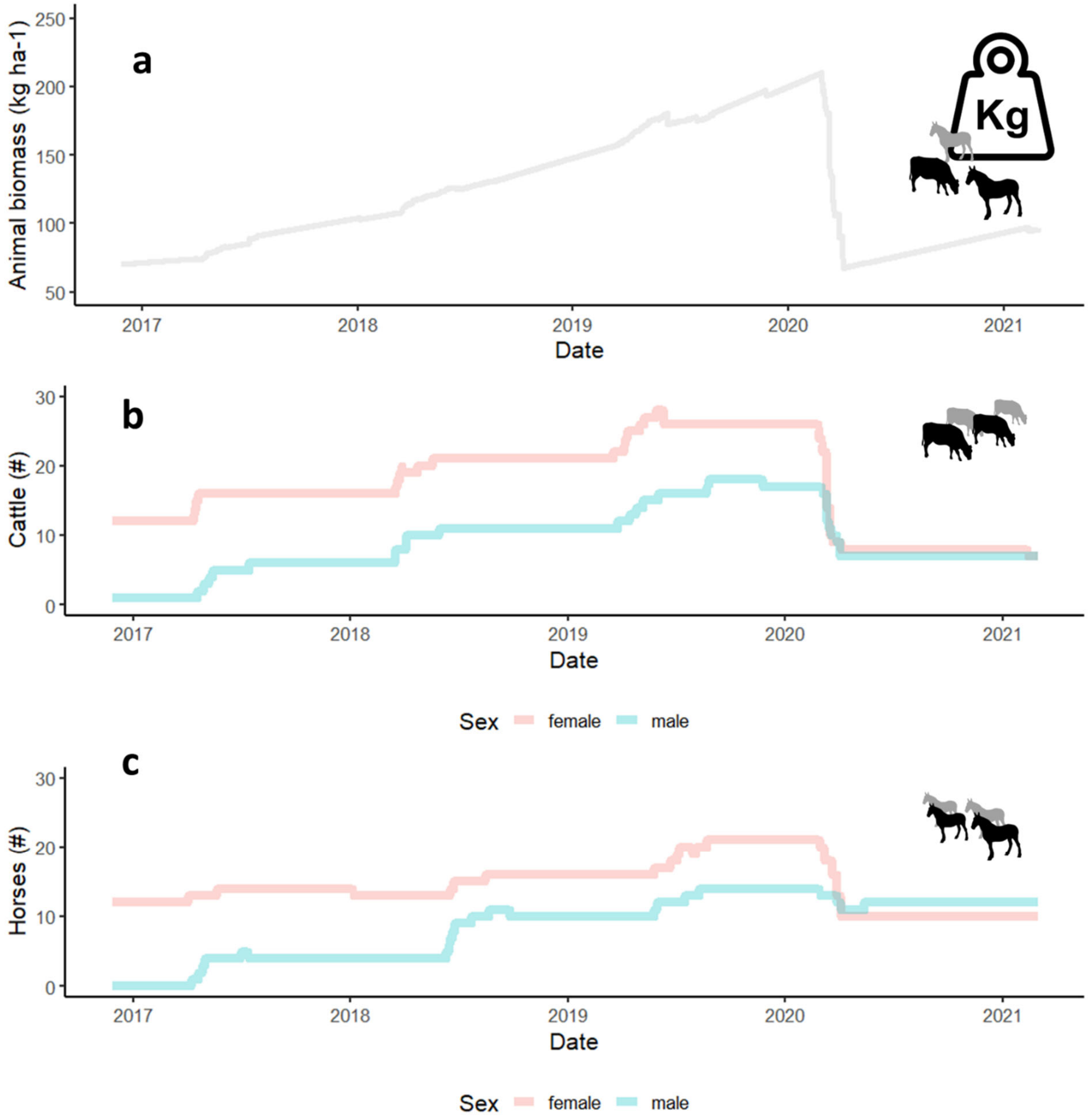


***Figure S2****: Total estimated animal biomass (a), number of cattle (b), and horses (c) during the time of GPS deployment. The following icons were used under CC BY-3.0 Attribution License from the Noun Project: Kg (Paulius B.). The following icons were used under CC0 1.0 Universal Public Domain Dedication from Phylopic: Cattle (Bos primigenius taurus Linnaeus 1758), Horse (Equus ferus caballus Linnaeus 1758).*

### *ALS-based vegetation height and density*

Vegetation heights for each pixel were represented as the maximum recorded height for a pixel rather than the mean, which makes the dataset highly sensitive to outliers. Rather than setting arbitrary class boundaries, we used a shiny app (https://popecology.shinyapps.io/shiny/) to iteratively explore natural breaks in vegetation heights. We arrived at the following classes: short: ≤0.5m, intermediate: >0.5 and ≤7m, and tall vegetation: >7m. When the fence-line cut through forested areas, trees were cut down to establish the outer fence prior to animal introduction in 2016. As we do not know exactly when this happened, we excluded data points falling within a 10m buffer zone around the outer fence prior to analyses.

We used the vegetation density dataset from (Assmann et al., 2022) as a predictor of animal preference. Despite ALS having a deeper penetration than passive spectral methods, such as NDVI, the signal still attenuates in dense canopies. This may lead to apparent, yet potentially false, low vegetation density in pixels with dense canopies. A potential implication is that certain shrubland-pixels may appear denser than forests in the dataset, simply because of the forest canopy attenuation of the ALS signal. However, this is mainly an issue for dense needle-leaved forests, as the broadleaved forests in the study area a relatively open. Furthermore, the ALS datasets for our specific study area were recorded during the winter (December and January) before or around budburst. For these reasons, we do not consider this a main caveat of this study.

### *Vegetation greenness measured as NDVI*

We obtained the Sentinel-2 NDVI for the growing season (1 April to 31 October) for each year since 2017, when the first full Sentinel-2 data was available for a full growing season, with the last year being 2022. After removing images with >50 % of cells covered by clouds and masking out pixels with detected cirrus and cloud formations, NDVI was calculated for each image as the normalized difference between near-infrared bands and red bands as NDVI=(NIR−Red)/(NIR+Red).

### *GPS collar data for movement analyses*

GPS fixes were obtained using the VERTEX Plus collar, which had an average accuracy of 8-15 metres (Vectronic Aerospace Gmbh, Berlin, Germany). We checked for precision of GPS fixes using the dilution of precision (DOP); all fixes had good precision (DOP<5).

One collar for horses was deployed only from July 2018, whereas both collars for cattle were removed in March 2019 and re-deployed in June 2019. All collars were removed from March to October 2020 to download other data that was not used in this study. In total, we had 150,173 GPS fixes (Table S1, Figure S3).

***Table S1:*** *Summary of the GPS collar data.*

| Population | CollarID | n | start of data transmission | end of data transmission | missing periods |
|---|---|---|---|---|---|
| **Exmoor (horse)** | 27891 | 44253 | December 2017 | March 2021 | April 2020 - September 2020 |
| **Exmoor (horse)** | 27893 | 35529 | July 2018 | March 2021 | March 2020 - September 2020 |

| | | | | | |
|---|---|---|---|---|---|
| **Galloway (cattle)** | 27890 | 39195 | December 2017 | March 2021 | March 2019 - June 2019 and March 2020 - September 2020 |
| **Galloway (cattle)** | 27892 | 31196 | December 2017 | March 2021 | March 2019 - June 2019 and March 2020 - September 2020 |

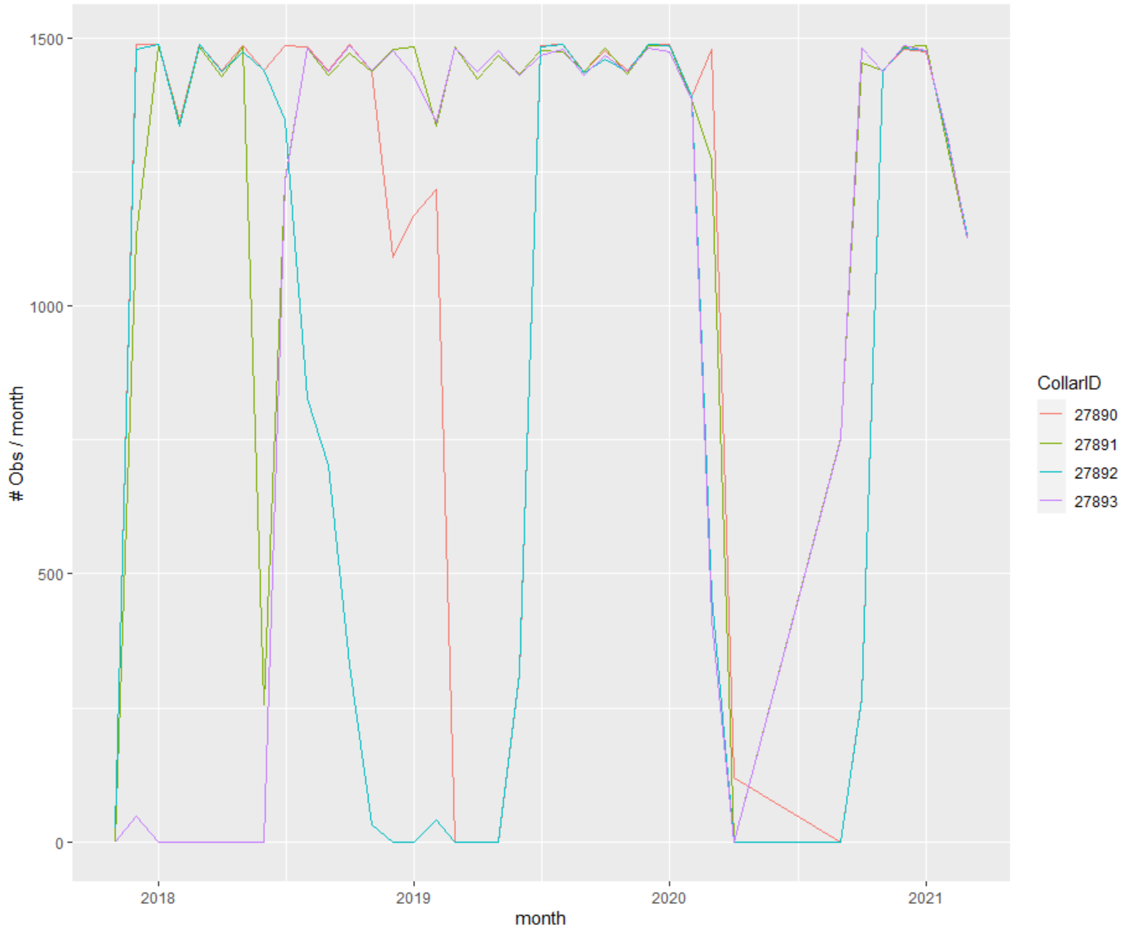


***Figure S3**: Temporal distribution of the GPS fixes*

## *State-space modelling*

The state-space model decomposed the movement process into different behavioural states and associated movement parameters (Jonsen et al. 2013). We decomposed the movement process into distinct underlying states using a hidden Markov model approach (HMM; Jonsen et al., 2013; Michelot et al., 2016). HMMs are a class of state-space models that describe animal behaviour as a set of states defined by movement parameters and a matrix of probabilities of transitions among states (Jonsen et al. 2005). Each state is characterised by its movement parameters, e.g. states associated with long-distance dispersal have higher average step lengths. We fitted HMMs to cattle and horses GPS data specifying a Gamma distribution (Γ) for step lengths and a Von Mises distribution (VM) for turning angles. Both distributions require an expected value (L for step length and θ for turning angle) and a measure of dispersion, namely standard deviation for step length ($\sigma_L$) and concentration for turning angles (κ). Cattle and horses were analysed separately, as the two species have different movement characteristics.

Fitting HMMs requires a pre-defined number of movement states and initial distribution parameters. This may influence HMM results, as different numbers of states can lead to different parameter estimates and fit using different starting parameters can diverge (Michelot et al. 2016). In order to use realistic values for the number of states and initial parameters, we explored the empirical distributions of step lengths and turning angles derived from GPS data (Figure S4).

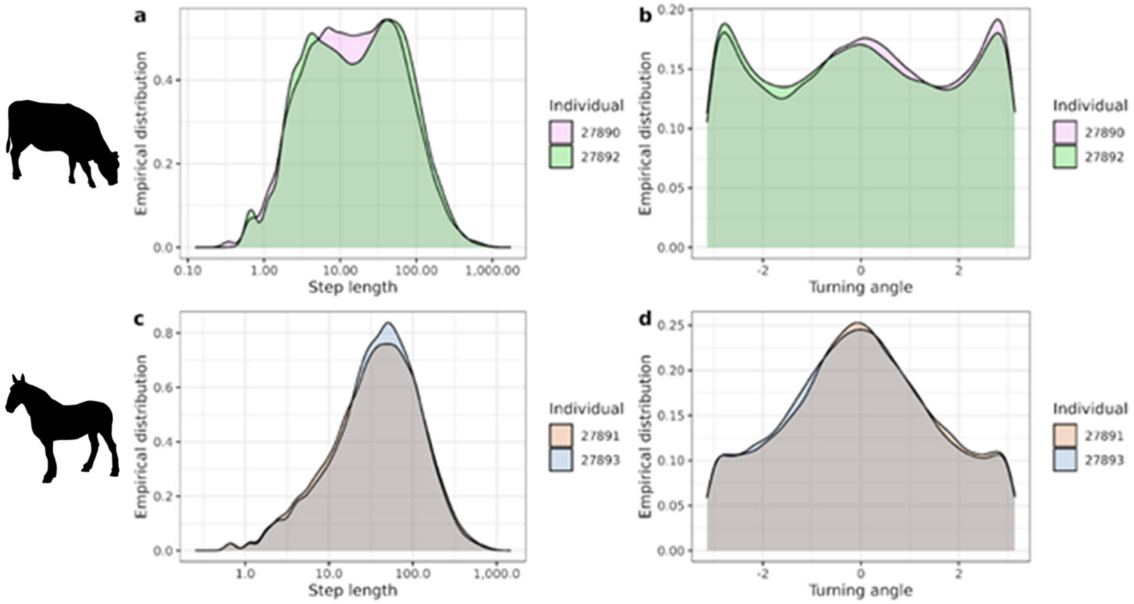


***Figure S4***: *Empirical distribution of step lengths (a,c) and turning angles (b,d) for cattle (a,b) and horses (c,d ). The following icons were used under CC0 1.0 Universal Public Domain Dedication from Phylopic: Cattle (Bos primigenius taurus Linnaeus 1758), Horse (Equus ferus caballus Linnaeus 1758).*

In particular: we chose a set of numbers of states that reflected the numbers of local maxima of the empirical distributions; and defined uniform distributions centered around the local maxima to inform the initial parameters. We fitted two sets of HMMs per animal, one with two movement states and one with three, using starting parameters drawn from uniform distributions centered around the empirical local maxima. We then compared HMMs with different numbers of states using AIC and retained only the most parsimonious ones. For each animal population, we replicated the fitting procedure for the chosen HMM 20 times in order to assess the sensitivity of results to initial starting conditions (Table S2).

***Table S2***: *Starting parameters for the most parsimonious HMMs.*

| **Population** | **Parameter** | **State 1** | **State 2** | **State 3** |
|---|---|---|---|---|
| **Cattle** | L | U(1,3) | U(3,15) | U(15,50) |
| | $\sigma_L$ | U(1,2) | U(4,15) | U(40,250) |
| | θ | U(2,3) | U(1,2) | U(0,0.5) |
| | κ | 1 | 1 | 1 |

| | | | | |
|---|---|---|---|---|
| **Horses** | L | U(1,10) | U(10,100) | - |
| | $\sigma_L$ | U(5,50) | U(5,50) | - |
| | θ | U(2,5,3,14) | U(0.1,2.5) | - |
| | κ | 1 | 1 | - |

We also allowed the probability of transition among states to vary with time of the day and period of the year as:

$$Hour.x = -cos\left(\frac{\pi}{12} * Hour\right)$$

$$Hour.y = sin\left(\frac{\pi}{6} * (Hour - 3)\right)$$

$$Month.x = -cos\left(\frac{\pi}{6} * (Month - 1.5)\right)$$

where Hour was the hour of the day and Month the calendar month. The rationale was to allow animals to preferentially choose a movement state depending on proximity to midday (Hour.x), hours around midday (Hour.y) and seasonality (Month.x) (Figure S5).

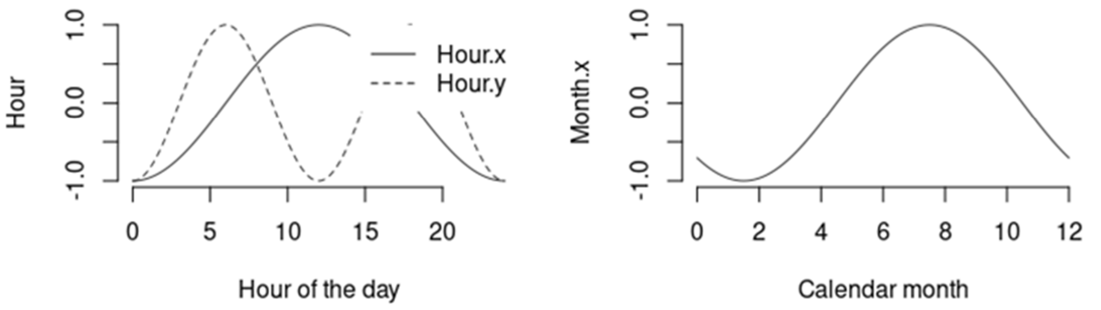


***Figure S5***: *Probability of transition among states depending on time of the day and period of the year.*

Cattle and horses were analyzed separately, as the two species have different movement capabilities. We fitted the HMMs using the R package *moveHMM* (Michelot et al. 2016).

For cattle, this approach suggested three states - resting, foraging, exploring – while it only decomposed horse movement into two behavioural states, namely resting/foraging and exploration. It is worth noting that the average movement distance per 30-minute timestep between GPS fixes is not necessarily the same for cattle and horses in the same state. Cattle moved on average 4, 22, and 83 m between GPS fixes in resting, foraging and exploratory states respectively. For horses, the values are 29 and 130 m for the resting/foraging and exploratory states.

## *Step-selection function*

To investigate animal preferences within the rewilding area, we assigned the respective behavioural states to the GPS fixes and used this to simulate random habitat preferences (Michelot et al. 2024). By

comparing these random expectations with observed preferences, we assessed how animals use the area. This approach has the advantage of explicitly taking into account how animals move at each time, sampling random expectations from areas that are immediately available for individuals (Karelus et al. 2019). More specifically, we used a conditional logistic regression (Craiu et al. 2011, Thurfjell et al. 2014, Karelus et al. 2019, Michelot et al. 2024). From the HMMs we obtained the fitted movement parameters, describing the distribution of step lengths and turning angles for each state and animal population, and the state of each GPS fix, obtained using the Viterbi algorithm (Zucchini et al. 2017). We used these fitted distributions (Figure S4) to draw six random points for each fix based on their movement state and associated movement parameters. For each fix, we drew six random values from the distributions of step length and turning angle of the respective state and used them to obtain six random locations at time t+1.

If an animal had a GPS fix (xt,yt)i at time t in movement state i, then six random points (xt+1,yt+1)i0 were generated for time t+1 as:

$$x^0_{(t+1)} = x_t + \Gamma(\mu_i, \sigma_i) \cdot cos\big(\alpha_t + VM(\theta_i, \kappa_i)\big)\ y^0_{(t+1)}$$
$$= y_t + \Gamma(\mu_i, \sigma_i) \cdot sin\big(\alpha_t + VM(\theta_i, \kappa_i)\big)$$

where VM(θ, κ) in [0, 2π], and α is the absolute angle of the movement between fix t−1 and t: $\alpha_t = atan\left(\frac{y_t - y_{t-1}}{x_t - x_{t-1}}\right)$.

These six random points were used as random expectations and compared with the observed value $(x_{t+1},y_{t+1})_i^1$ using a conditional logistic regression (Thurfjell et al. 2014, Karelus et al. 2019). Conditional logistic regression was performed using each observation (including the six random points) as a stratum and each animal as a cluster using the package *TwoStepCLogit* (Craiu et al. 2011).

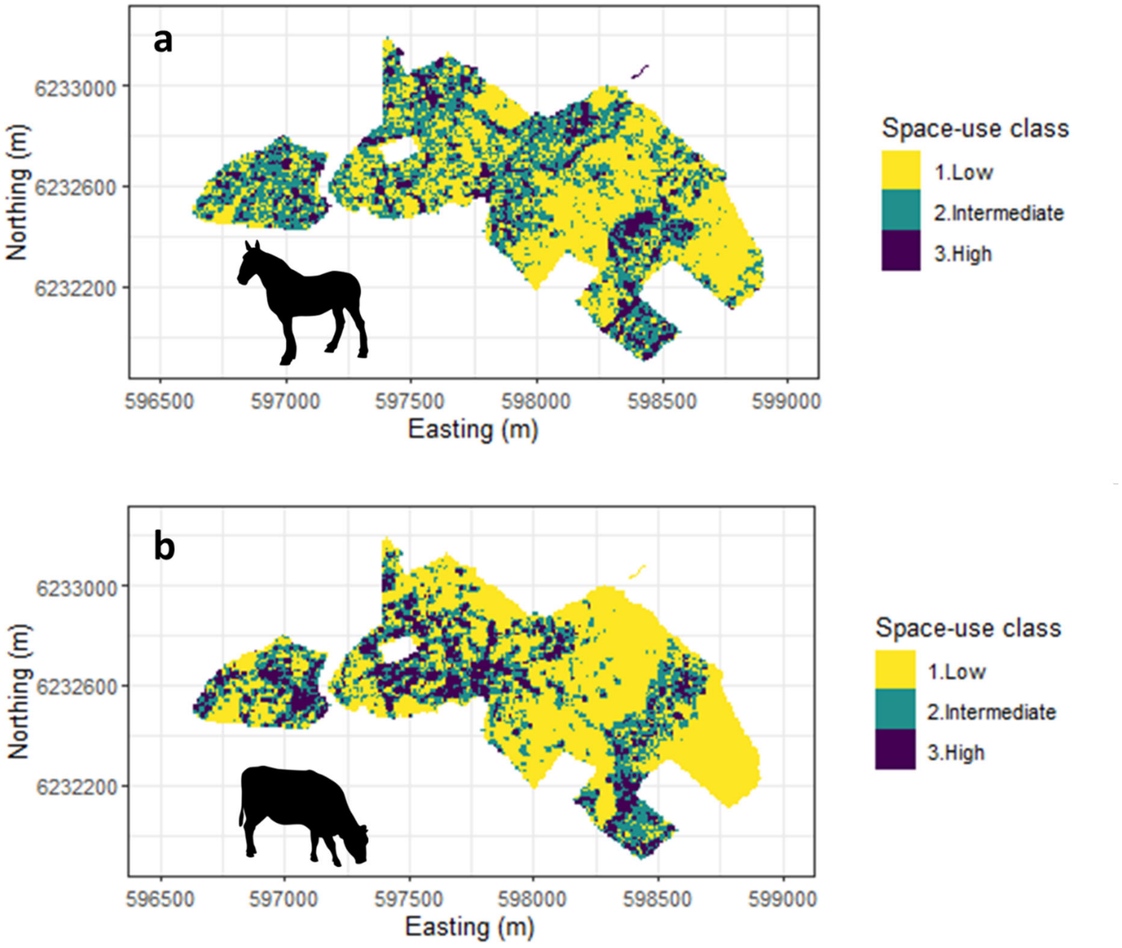


***Figure S6****: Horse (a) and cattle (b) space use classes (low: <4, intermediate: >4 and <12, high: >12 GPS fix counts per pixel). The following icons were used under CC0 1.0 Universal Public Domain Dedication from Phylopic: Cattle (Bos primigenius taurus Linnaeus 1758), Horse (Equus ferus caballus Linnaeus 1758).*

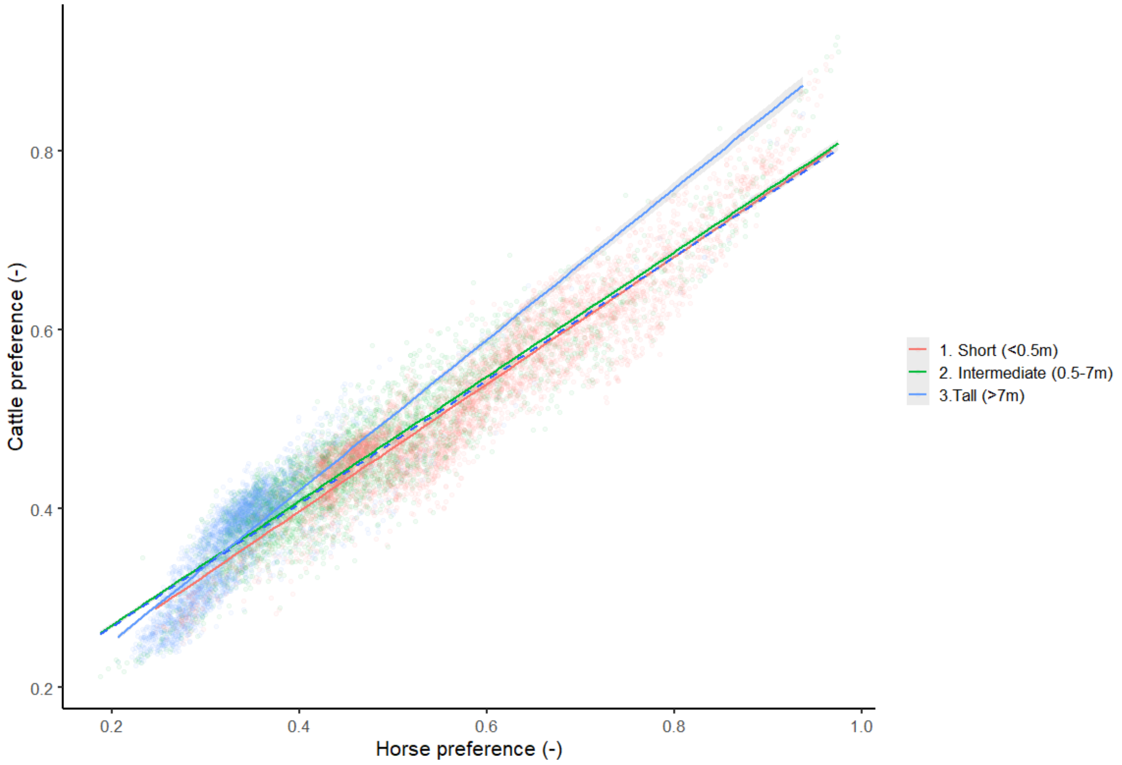


***Figure S7****: Cattle preference plotted against horse preference and linear regression lines fitted to all data (dashed), short (blue), intermediate (green) and short (orange) vegetation for visualisation. The shadings show the 95% confidence intervals. All fits are highly significant (p<0.001).*

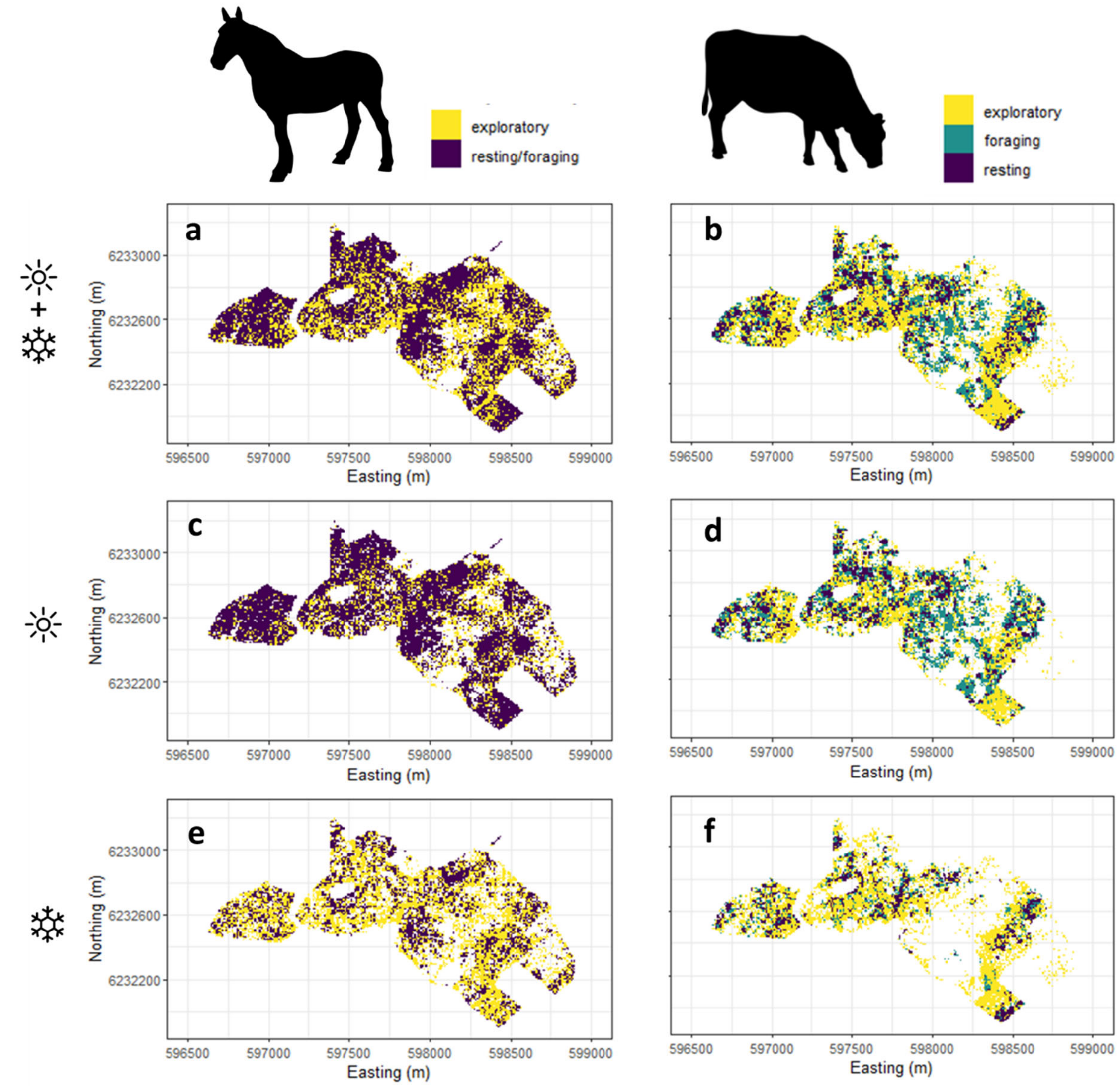


***Figure S8****: Maps of the dominating mobility states for horses (a,c,e) and cattle (b,d,f) across the entire year (a,b), summer (c,d) and winter (e,f). The following icons were used under CC BY-3.0 Attribution License from the Noun Project: Sun (Jajang Nurrahman), Snowflake (Focus Lab). The following icons were used under CC0 1.0 Universal Public Domain Dedication from Phylopic: Cattle (Bos primigenius taurus Linnaeus 1758), Horse (Equus ferus caballus Linnaeus 1758).*

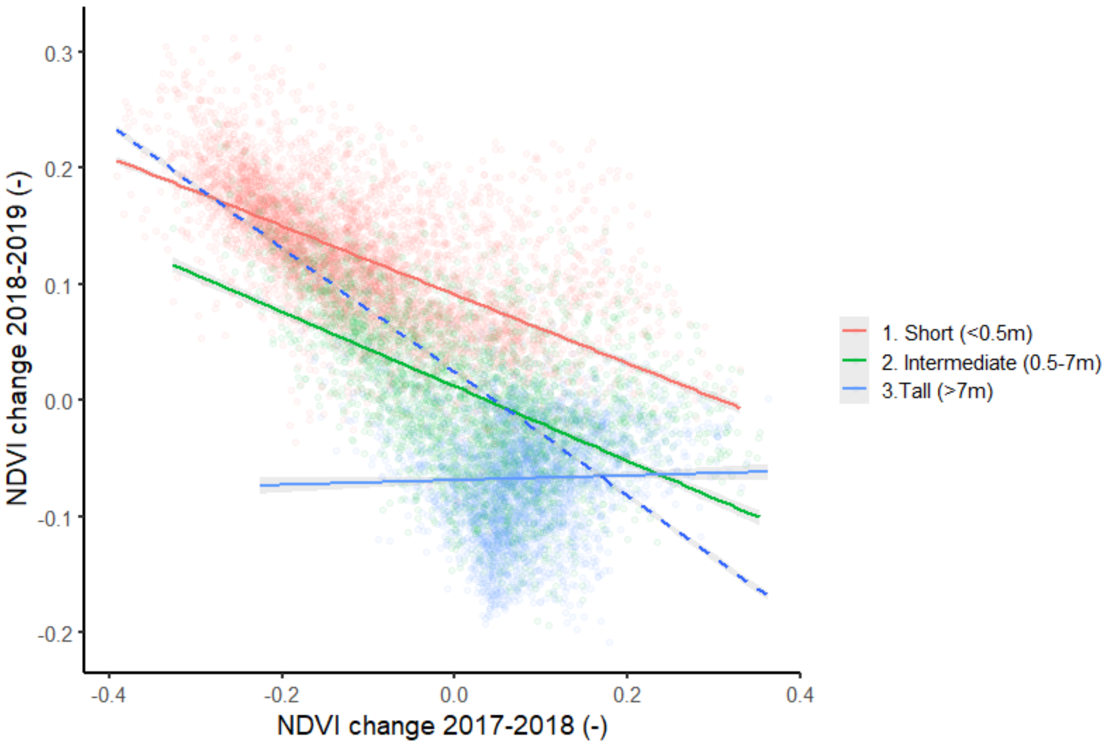


***Figure S9***: *NDVI recovery immediately after the drought (2018 to 2019) plotted agsinst the NDVI drop during the drought (2017-2018) and linear regression lines fitted to all data (dashed), short (blue), intermediate (green) and short (orange) vegetation for visualisation. The shadings show the 95% confidence intervals. All fits are highly significant (p<0.001). Decreasing proportions of the variation is explained by all>short>intermediate>>tall vegetation classes.*

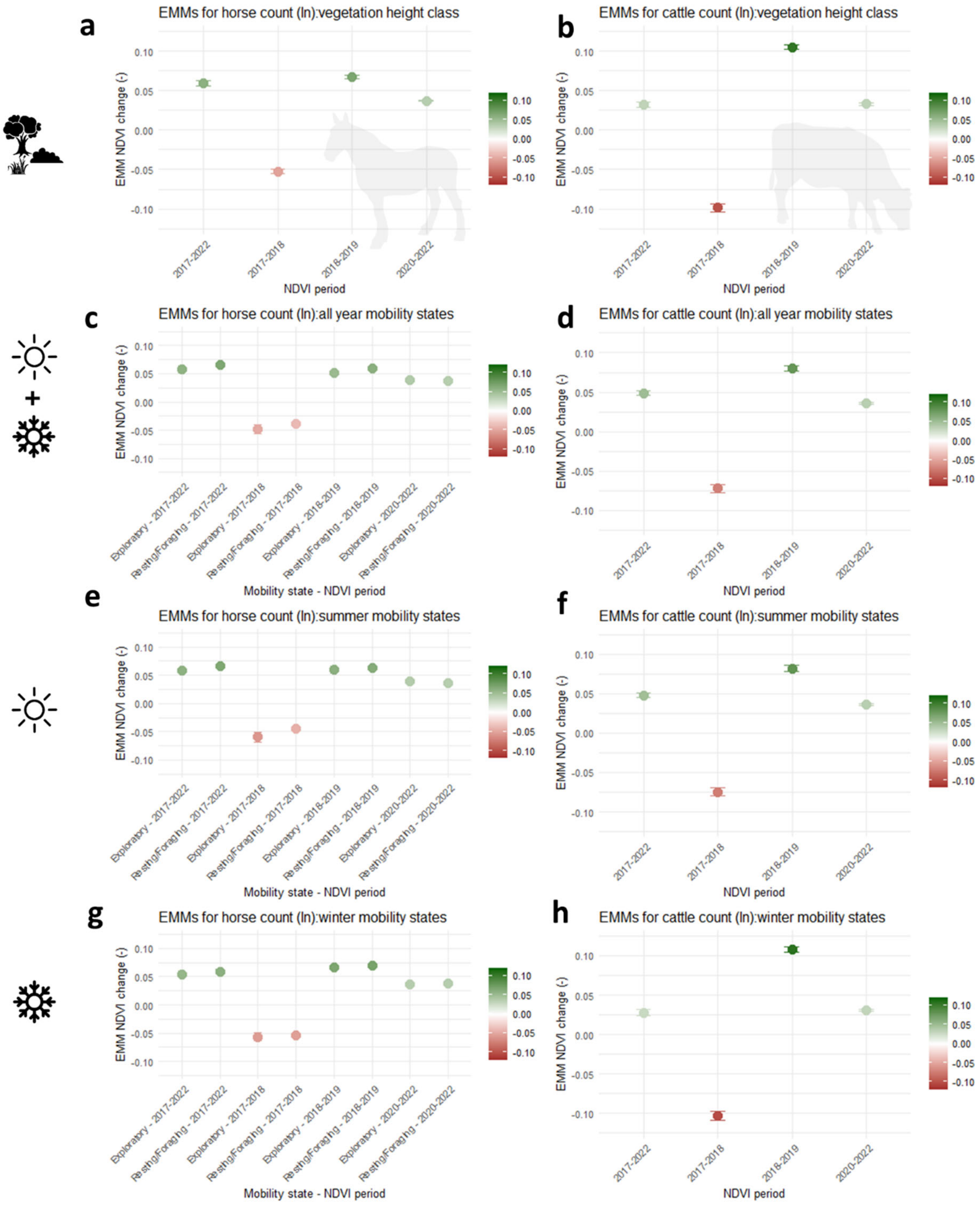


***Figure S10****: Estimated marginal mean NDVI change effects of horse (a,c,e,g) and cattle (b,d,f,h) count (ln) conditioned by vegetation height class (a,b) and the mobility states for all year (c,d), summer (e,f) and winter (g,h). For horses, different EMMs were estimated for each mobility state and period, while there was no difference between mobility states for cattle. The following icons were used under CC BY-3.0 Attribution License from the Noun Project: Sun (Jajang Nurrahman), Snowflake (Focus Lab). The following icons were used under CC0 1.0 Universal Public Domain Dedication from Phylopic: Cattle (Bos primigenius taurus Linnaeus 1758), Horse (Equus ferus caballus Linnaeus 1758).*

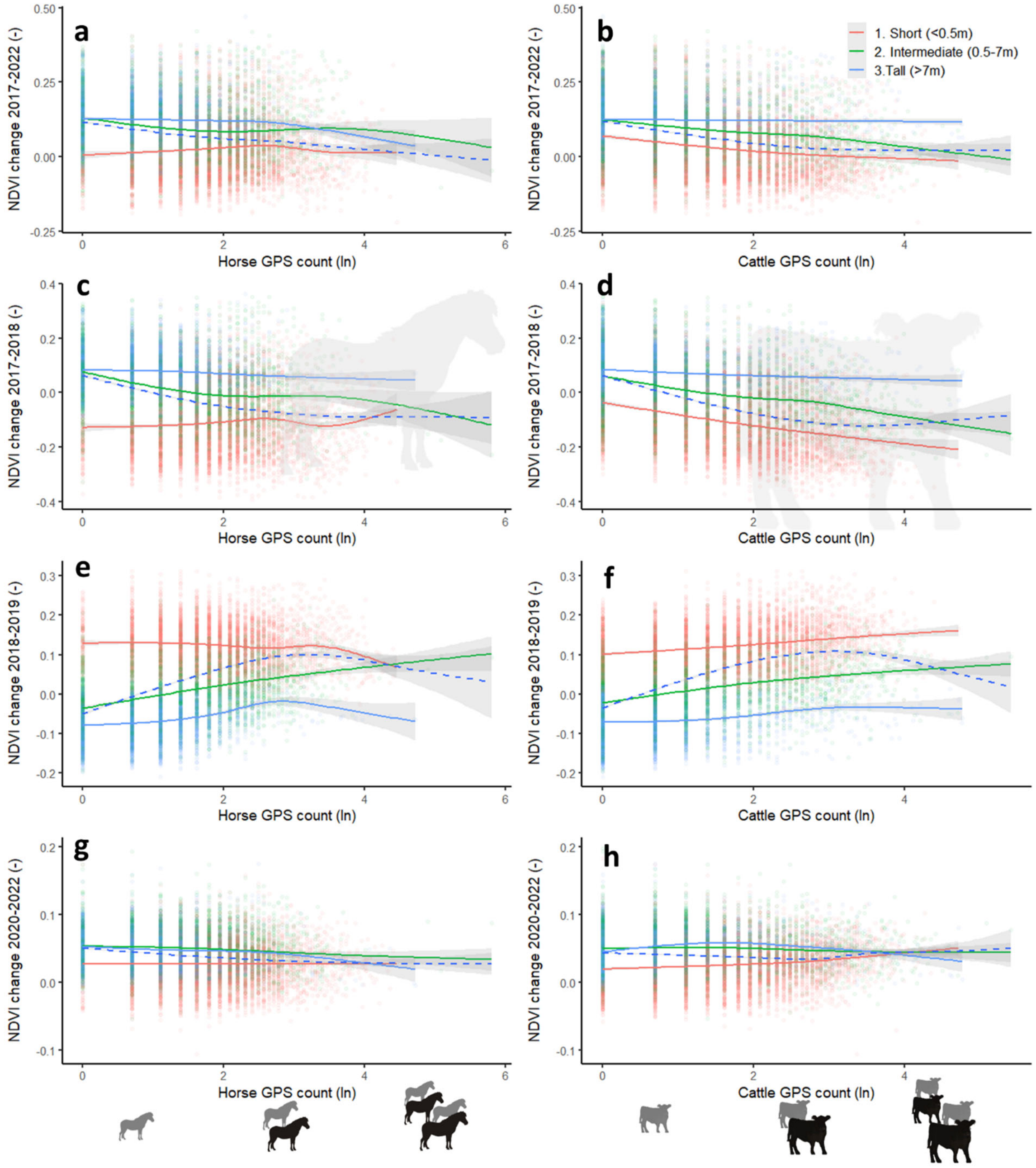


***Figure S11****: NDVI change from 2017 to 2022 (a,b), 2017-2018 (c,d) 2018-2019 (e,f) and 2020-2022 (g,h) plotted against horse (a,c,e) and cattle (b,d,f) space use. The trend lines show GAM-smoothed curves for all data (dashed), short (orange), intermediate (green) and tall (blue) vegetation. The shadings show the 95% confidence intervals. See Table S3 for model fits. The following icons were used under CC0 1.0 Universal Public Domain Dedication from Phylopic: Cattle (Bos primigenius taurus Linnaeus 1758), Horse (Equus ferus caballus Linnaeus 1758).*

***Table S3:*** *Generalised additive model summaries. All variables with a p<0.05 are considered significant. Variables in grey are insignificant (p>0.05).*

| Predicted | Animal | Mobility state | Model | $R^2_{adj}$ | obs | AIC | BIC | Fixed term 1 (Intercept) | | | | Smooth term 1 | | | | Smooth term 2 | | | | Smooth term 3 | | | |
|---|---|---|---|---|---|---|---|---|---|---|---|---|---|---|---|---|---|---|---|---|---|---|---|
| | | | | | | | | Estimate | Std. Error | t value | Pr(>\|t\|) | edf | Ref.df | F | p-value | edf | Ref.df | F | p-value | edf | Ref.df | F | p-value |
| NDVI change 2017-2022 | | | NDVI2017_2022~ s(VegH_15) | 0.19 | 11491 | -22398 | -22323 | 0.075 | 0.001 | 87.90 | 0.00 | 8.21 | 8.83 | 309.71 | 0.00 | | | | | | | | |
| | Horses | | NDVI2017_2022~ s(ln.horse.count) | 0.05 | 11491 | -20535 | -20472 | 0.078 | 0.001 | 85.01 | 0.00 | 6.57 | 7.32 | 82.95 | 0.00 | | | | | | | | |
| | Horses | | NDVI2017_2022~ s(ln.horse.count:VegCl) | 0.17 | 11491 | -22144 | -22080 | -0.017 | 0.002 | -7.39 | 0.00 | 6.67 | 7.45 | 325.83 | 0.00 | | | | | | | | |
| | Cattle | | NDVI2017_2022~ s(ln.cattle.count) | 0.12 | 11491 | -21435 | -21393 | 0.078 | 0.001 | 88.36 | 0.00 | 3.69 | 4.47 | 354.35 | 0.00 | | | | | | | | |
| | Cattle | | NDVI2017_2022~ s(ln.cattle.count:VegCl) | 0.18 | 11491 | -22292 | -22252 | -0.008 | 0.002 | -3.30 | 0.00 | 3.41 | 3.99 | 652.01 | 0.00 | | | | | | | | |
| | Horses | | NDVI2017_2022~ s(VegH_15) + s(ln.horse.count) | 0.19 | 11491 | -22429 | -22303 | 0.075 | 0.001 | 88.05 | 0.00 | 8.28 | 8.86 | 233.69 | 0.00 | 6.85 | 7.58 | 5.48 | 0.00 | | | | |
| | Cattle | | **NDVI2017_2022~ s(VegH_15) +s(ln.cattle.count)** | **0.22** | **11491** | **-22748** | **-22667** | 0.075 | 0.001 | 89.25 | 0.00 | 8.01 | 8.74 | 167.38 | 0.00 | 1.00 | 1.00 | 358.67 | 0.00 | | | | |
| | Both | | NDVI2017_2022~ s(ln.horse.count)+s(ln.cattle.count) | 0.13 | 11491 | -21531 | -21441 | 0.078 | 0.001 | 88.94 | 0.00 | 6.61 | 7.36 | 14.68 | 0.00 | 3.70 | 4.47 | 233.53 | 0.00 | | | | |
| | Both | | **NDVI2017_2022~ s(VegH_15) +s(ln.horse.count)+s(ln.cattle.count)** | **0.22** | **11491** | **-22773** | **-22642** | 0.075 | 0.001 | 89.38 | 0.00 | 8.05 | 8.76 | 155.99 | 0.00 | 6.83 | 7.56 | 4.95 | 0.00 | 1.00 | 1.00 | 354.18 | 0.00 |
| | Cattle | All year | NDVI2017_2022~ s(VegH_15) +s(ln.cattle.count:MobState_all_year) | 0.18 | 7988 | -15159 | -15065 | 0.070 | 0.003 | 21.37 | 0.00 | 7.54 | 8.46 | 126.18 | 0.00 | 3.91 | 4.59 | 41.51 | 0.00 | | | | |
| | Cattle | Summer | NDVI2017_2022~ s(VegH_15) +s(ln.cattle.count:MobState_summer) | 0.18 | 7030 | -13382 | -13305 | 0.070 | 0.004 | 19.77 | 0.00 | 6.55 | 7.68 | 116.77 | 0.00 | 2.77 | 3.23 | 51.87 | 0.00 | | | | |
| | Cattle | Winter | NDVI2017_2022~ s(VegH_15) +s(ln.cattle.count:MobState_winter) | 0.15 | 5233 | -10009 | -9925 | 0.040 | 0.004 | 9.37 | 0.00 | 6.99 | 8.06 | 83.06 | 0.00 | 3.89 | 4.60 | 14.70 | 0.00 | | | | |
| | Horses | All year | NDVI2017_2022~ s(VegH_15) +s(ln.horses.count:MobState_all_year) | 0.18 | 9850 | -18853 | -18709 | 0.078 | 0.002 | 39.23 | 0.00 | 8.41 | 8.90 | 201.91 | 0.00 | 9.60 | 9.91 | 5.75 | 0.00 | | | | |
| | Horses | Summer | NDVI2017_2022~ s(VegH_15) +s(ln.horses.count:MobState_summer) | 0.16 | 8769 | -16506 | -16381 | 0.074 | 0.002 | 35.83 | 0.00 | 7.95 | 8.71 | 168.84 | 0.00 | 7.69 | 8.51 | 4.56 | 0.00 | | | | |
| | Horses | Winter | NDVI2017_2022~ s(VegH_15) +s(ln.horses.count:MobState_winter) | 0.16 | 7401 | -14086 | -13954 | 0.065 | 0.003 | 23.77 | 0.00 | 8.05 | 8.76 | 135.00 | 0.00 | 8.97 | 9.49 | 3.02 | 0.00 | | | | |
| NDVI change 2017-2018 | | | NDVI2017_2018~ s(VegH_15) | 0.39 | 11491 | -19043 | -18965 | -0.020 | 0.001 | -20.42 | 0.00 | 8.71 | 8.97 | 810.81 | 0.00 | | | | | | | | |
| | Horses | | NDVI2017_2018~ s(ln.horse.count) | 0.11 | 11491 | -14769 | -14702 | -0.020 | 0.001 | -16.95 | 0.00 | 7.20 | 7.89 | 183.87 | 0.00 | | | | | | | | |
| | Horses | | NDVI2017_2018~ s(ln.horse.count:VegCl) | 0.36 | 11491 | -18534 | -18460 | -0.192 | 0.003 | -71.97 | 0.00 | 8.07 | 8.79 | 735.54 | 0.00 | | | | | | | | |
| | Cattle | | NDVI2017_2018~ s(ln.cattle.count) | 0.25 | 11491 | -16644 | -16593 | -0.020 | 0.001 | -18.39 | 0.00 | 4.93 | 5.76 | 647.98 | 0.00 | | | | | | | | |
| | Cattle | | NDVI2017_2018~ s(ln.cattle.count:VegCl) | 0.38 | 11491 | -18977 | -18921 | -0.176 | 0.003 | -66.54 | 0.00 | 5.61 | 6.43 | 1115.14 | 0.00 | | | | | | | | |
| | Horses | | NDVI2017_2018~ s(VegH_15) + s(ln.horse.count) | 0.39 | 11491 | -19109 | -18971 | -0.020 | 0.001 | -20.48 | 0.00 | 8.74 | 8.98 | 588.35 | 0.00 | 7.99 | 8.55 | 9.10 | 0.00 | | | | |
| | Cattle | | **NDVI2017_2018~ s(VegH_15) +s(ln.cattle.count)** | **0.43** | **11491** | **-19903** | **-19816** | -0.020 | 0.001 | -21.20 | 0.00 | 8.63 | 8.96 | 439.50 | 0.00 | 1.20 | 1.37 | 618.72 | 0.00 | | | | |
| | Both | | NDVI2017_2018~ s(ln.horse.count)+s(ln.cattle.count) | 0.27 | 11491 | -16946 | -16839 | -0.020 | 0.001 | -18.64 | 0.00 | 7.33 | 8.01 | 39.72 | 0.00 | 5.20 | 6.03 | 398.60 | 0.00 | | | | |
| | Both | | **NDVI2017_2018~ s(VegH_15) +s(ln.horse.count)+s(ln.cattle.count)** | **0.43** | **11491** | **-19942** | **-19795** | -0.020 | 0.001 | -21.24 | 0.00 | 8.67 | 8.97 | 391.02 | 0.00 | 7.60 | 8.24 | 6.19 | 0.00 | 1.61 | 2.00 | 432.13 | 0.00 |
| | Cattle | All year | NDVI2017_2018~ s(VegH_15) +s(ln.cattle.count:MobState_all_year) | 0.35 | 7988 | -12942 | -12840 | -0.052 | 0.004 | -13.75 | 0.00 | 8.47 | 8.92 | 320.22 | 0.00 | 4.17 | 4.89 | 73.65 | 0.00 | | | | |
| | Cattle | Summer | NDVI2017_2018~ s(VegH_15) +s(ln.cattle.count:MobState_summer) | 0.36 | 7030 | -11503 | -11398 | -0.047 | 0.004 | -11.62 | 0.00 | 8.22 | 8.83 | 271.22 | 0.00 | 5.12 | 5.94 | 60.00 | 0.00 | | | | |
| | Cattle | Winter | NDVI2017_2018~ s(VegH_15) +s(ln.cattle.count:MobState_winter) | 0.29 | 5233 | -8216 | -8136 | -0.116 | 0.005 | -22.95 | 0.00 | 8.18 | 8.82 | 176.30 | 0.00 | 2.00 | 2.00 | 94.87 | 0.00 | | | | |
| | Horses | All year | NDVI2017_2018~ s(VegH_15) +s(ln.horses.count:MobState_all_year) | 0.36 | 9850 | -15955 | -15857 | -0.021 | 0.002 | -9.04 | 0.00 | 8.76 | 8.98 | 522.01 | 0.00 | 2.96 | 3.45 | 12.28 | 0.00 | | | | |
| | Horses | Summer | NDVI2017_2018~ s(VegH_15) +s(ln.horses.count:MobState_summer) | 0.34 | 8769 | -13956 | -13816 | -0.026 | 0.002 | -10.87 | 0.00 | 8.73 | 8.98 | 422.13 | 0.00 | 8.95 | 9.55 | 6.61 | 0.00 | | | | |
| | Horses | Winter | NDVI2017_2018~ s(VegH_15) +s(ln.horses.count:MobState_winter) | 0.34 | 7401 | -11758 | -11623 | -0.045 | 0.003 | -14.11 | 0.00 | 8.63 | 8.96 | 354.19 | 0.00 | 8.95 | 9.48 | 3.55 | 0.00 | | | | |
| NDVI change 2018-2019 | | | NDVI2018_2019~ s(VegH_15) | 0.70 | 11491 | -34101 | -34021 | 0.035 | 0.001 | 68.30 | 0.00 | 8.98 | 9.00 | 3028.71 | 0.00 | | | | | | | | |
| | Horses | | NDVI2018_2019~ s(ln.horse.count) | 0.22 | 11491 | -22933 | -22870 | 0.035 | 0.001 | 42.01 | 0.00 | 6.60 | 7.36 | 430.89 | 0.00 | | | | | | | | |
| | Horses | | NDVI2018_2019~ s(ln.horse.count:VegCl) | 0.66 | 11491 | -32573 | -32486 | 0.205 | 0.001 | 141.35 | 0.00 | 9.77 | 9.97 | 2249.74 | 0.00 | | | | | | | | |
| | Cattle | | NDVI2018_2019~ s(ln.cattle.count) | 0.29 | 11491 | -24092 | -24040 | 0.035 | 0.001 | 44.18 | 0.00 | 5.20 | 6.03 | 782.57 | 0.00 | | | | | | | | |
| | Cattle | | NDVI2018_2019~ s(ln.cattle.count:VegCl) | 0.67 | 11491 | -32759 | -32705 | 0.202 | 0.001 | 139.20 | 0.00 | 5.39 | 6.21 | 3701.88 | 0.00 | | | | | | | | |
| | Horses | | NDVI2018_2019~ s(VegH_15) + s(ln.horse.count) | 0.71 | 11491 | -34317 | -34172 | 0.035 | 0.001 | 68.97 | 0.00 | 8.92 | 9.00 | 2163.39 | 0.00 | 8.74 | 8.96 | 25.81 | 0.00 | | | | |
| | Cattle | | **NDVI2018_2019~ s(VegH_15) +s(ln.cattle.count)** | **0.72** | **11491** | **-34747** | **-34650** | 0.035 | 0.000 | 70.25 | 0.00 | 8.98 | 9.00 | 1983.29 | 0.00 | 2.20 | 2.75 | 242.17 | 0.00 | | | | |
| | Both | | NDVI2018_2019~ s(ln.horse.count)+s(ln.cattle.count) | 0.36 | 11491 | -25264 | -25153 | 0.035 | 0.001 | 46.50 | 0.00 | 7.37 | 8.04 | 155.05 | 0.00 | 5.67 | 6.48 | 399.83 | 0.00 | | | | |
| | Both | | **NDVI2018_2019~ s(VegH_15) +s(ln.horse.count)+s(ln.cattle.count)** | **0.72** | **11491** | **-34875** | **-34712** | 0.035 | 0.000 | 70.67 | 0.00 | 8.93 | 9.00 | 1687.50 | 0.00 | 8.76 | 8.97 | 16.14 | 0.00 | 2.49 | 3.10 | 185.59 | 0.00 |
| | Cattle | All year | NDVI2018_2019~ s(VegH_15) +s(ln.cattle.count:MobState_all_year) | 0.66 | 7988 | -23451 | -23358 | 0.070 | 0.002 | 36.20 | 0.00 | 8.71 | 8.98 | 1350.55 | 0.00 | 2.50 | 2.85 | 84.45 | 0.00 | | | | |
| | Cattle | Summer | NDVI2018_2019~ s(VegH_15) +s(ln.cattle.count:MobState_summer) | 0.66 | 7030 | -20731 | -20641 | 0.066 | 0.002 | 31.76 | 0.00 | 8.65 | 8.96 | 1158.09 | 0.00 | 2.52 | 2.88 | 92.16 | 0.00 | | | | |
| | Cattle | Winter | NDVI2018_2019~ s(VegH_15) +s(ln.cattle.count:MobState_winter) | 0.55 | 5233 | -15460 | -15365 | 0.119 | 0.003 | 46.96 | 0.00 | 8.60 | 8.96 | 547.38 | 0.00 | 3.88 | 4.59 | 56.76 | 0.00 | | | | |
| | Horses | All year | NDVI2018_2019~ s(VegH_15) +s(ln.horses.count:MobState_all_year) | 0.68 | 9850 | -29180 | -29033 | 0.051 | 0.001 | 43.14 | 0.00 | 8.98 | 9.00 | 1913.36 | 0.00 | 9.49 | 9.86 | 10.45 | 0.00 | | | | |
| | Horses | Summer | NDVI2018_2019~ s(VegH_15) +s(ln.horses.count:MobState_summer) | 0.67 | 8769 | -26011 | -25865 | 0.055 | 0.001 | 45.20 | 0.00 | 8.94 | 9.00 | 1593.87 | 0.00 | 9.72 | 9.96 | 9.31 | 0.00 | | | | |
| | Horses | Winter | NDVI2018_2019~ s(VegH_15) +s(ln.horses.count:MobState_winter) | 0.66 | 7401 | -21798 | -21663 | 0.064 | 0.002 | 39.21 | 0.00 | 8.88 | 9.00 | 1341.52 | 0.00 | 8.69 | 9.27 | 8.21 | 0.00 | | | | |
| NDVI change 2020-2022 | | | NDVI2020_2022~ s(VegH_15) | 0.15 | 11491 | -48188 | -48111 | 0.040 | 0.000 | 144.38 | 0.00 | 8.45 | 8.91 | 222.77 | 0.00 | | | | | | | | |
| | Horses | | NDVI2020_2022~ s(ln.horse.count) | 0.03 | 11491 | -46764 | -46733 | 0.040 | 0.000 | 135.68 | 0.00 | 2.22 | 2.78 | 147.94 | 0.00 | | | | | | | | |
| | Horses | | NDVI2020_2022~ s(ln.horse.count:VegCl) | 0.10 | 11491 | -47530 | -47481 | 0.019 | 0.001 | 25.01 | 0.00 | 4.62 | 5.37 | 230.03 | 0.00 | | | | | | | | |
| | Cattle | | NDVI2020_2022~ s(ln.cattle.count) | 0.01 | 11491 | -46506 | -46458 | 0.040 | 0.000 | 134.17 | 0.00 | 4.50 | 5.33 | 27.68 | 0.00 | | | | | | | | |
| | Cattle | | NDVI2020_2022~ s(ln.cattle.count:VegCl) | 0.11 | 11491 | -47655 | -47598 | 0.015 | 0.001 | 19.71 | 0.00 | 5.73 | 6.55 | 210.08 | 0.00 | | | | | | | | |
| | Horses | | NDVI2020_2022~ s(VegH_15) + s(ln.horse.count) | 0.15 | 11491 | -48207 | -48119 | 0.040 | 0.000 | 144.51 | 0.00 | 8.45 | 8.91 | 173.63 | 0.00 | 1.51 | 1.87 | 12.16 | 0.00 | | | | |
| | Cattle | | NDVI2020_2022~ s(VegH_15) +s(ln.cattle.count) | **0.16** | **11491** | **-48294** | **-48180** | 0.040 | 0.000 | 145.09 | 0.00 | 8.46 | 8.92 | 218.00 | 0.00 | 5.07 | 5.90 | 19.12 | 0.00 | | | | |
| | Both | | NDVI2020_2022~ s(ln.horse.count)+s(ln.cattle.count) | 0.04 | 11491 | -46821 | -46759 | 0.040 | 0.000 | 136.04 | 0.00 | 2.11 | 2.66 | 121.14 | 0.00 | 4.41 | 5.24 | 11.43 | 0.00 | | | | |
| | Both | | NDVI2020_2022~ s(VegH_15) +s(ln.horse.count)+s(ln.cattle.count) | **0.16** | **11491** | **-48341** | **-48215** | 0.040 | 0.000 | 145.39 | 0.00 | 8.48 | 8.92 | 183.36 | 0.00 | 1.84 | 2.32 | 21.35 | 0.00 | 4.91 | 5.74 | 24.50 | 0.00 |
| | Cattle | All year | NDVI2020_2022~ s(VegH_15) +s(ln.cattle.count:MobState_all_year) | 0.16 | 7988 | -32975 | -32865 | 0.041 | 0.001 | 38.49 | 0.00 | 7.83 | 8.64 | 151.62 | 0.00 | 5.98 | 6.75 | 5.27 | 0.00 | | | | |
| | Cattle | Summer | NDVI2020_2022~ s(VegH_15) +s(ln.cattle.count:MobState_summer) | 0.16 | 7030 | -29165 | -29060 | 0.041 | 0.001 | 35.84 | 0.00 | 7.37 | 8.35 | 131.59 | 0.00 | 5.94 | 6.77 | 3.81 | 0.00 | | | | |
| | Cattle | Winter | NDVI2020_2022~ s(VegH_15) +s(ln.cattle.count:MobState_winter) | 0.11 | 5233 | -21649 | -21558 | 0.039 | 0.001 | 28.08 | 0.00 | 7.19 | 8.22 | 66.70 | 0.00 | 4.65 | 5.44 | 9.66 | 0.00 | | | | |
| | Horses | All year | NDVI2020_2022~ s(VegH_15) +s(ln.horses.count:MobState_all_year) | 0.14 | 9850 | -40939 | -40850 | 0.037 | 0.001 | 56.99 | 0.00 | 8.43 | 8.91 | 160.08 | 0.00 | 2.00 | 2.00 | 3.65 | 0.03 | | | | |
| | Horses | Summer | NDVI2020_2022~ s(VegH_15) +s(ln.horses.count:MobState_summer) | 0.13 | 8769 | -36278 | -36191 | 0.037 | 0.001 | 54.65 | 0.00 | 8.35 | 8.88 | 139.59 | 0.00 | 2.00 | 2.00 | 2.88 | 0.06 | | | | |
| | Horses | Winter | NDVI2020_2022~ s(VegH_15) +s(ln.horses.count:MobState_winter) | 0.13 | 7401 | -30654 | -30570 | 0.038 | 0.001 | 42.46 | 0.00 | 8.06 | 8.77 | 112.01 | 0.00 | 2.00 | 2.00 | 2.73 | 0.07 | | | | |

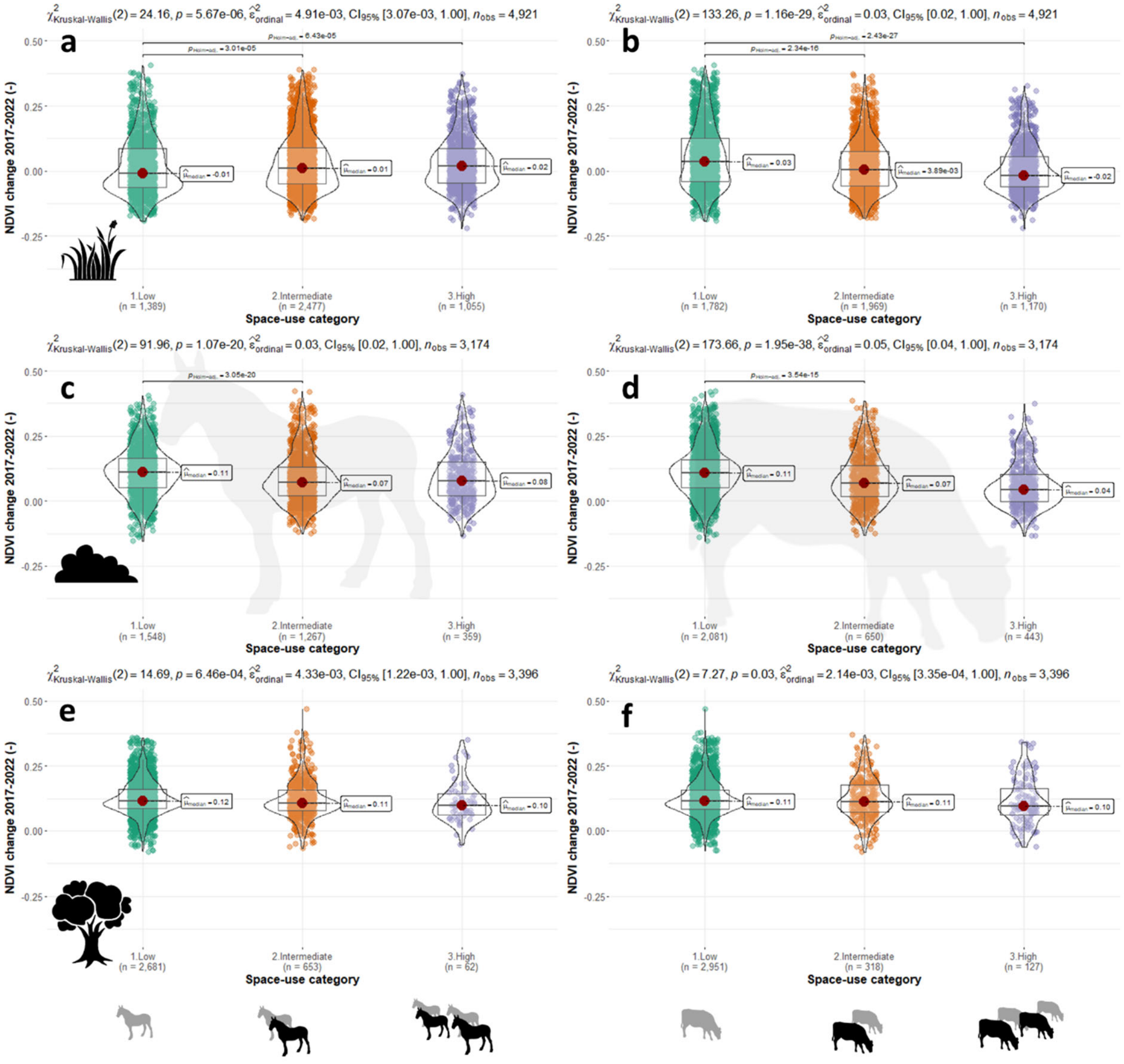


***Figure S12**: NDVI change since the inititiation of the rewilding project (2017-2022) for the three space-use categories (low: <4, intermediate: >4 and <12, high: >12 GPS fix counts per pixel) for horses (a,c,e) and cattle (b,d,f) for short (a,b), intermediate (c,d) and tall (e,f) vegetation. The horizontal line and red dot shows the median, the box edges show quartiles, and the violin plot shows the kernel density distributions of the data. P-values above the plot shows the results of the Kruskal-Wallis test, and the p-values for the pairwise comparisons (Dunn-tests) are shown when significant. The following icons were used under CC BY-3.0 Attribution License from the Noun Project: Grass (Nanik haq), Shrub (wahab marhaban), Tree (kareemov). The following icons were used under CC0 1.0 Universal Public Domain Dedication from Phylopic: Cattle (Bos primigenius taurus Linnaeus 1758), Horse (Equus ferus caballus Linnaeus 1758).*

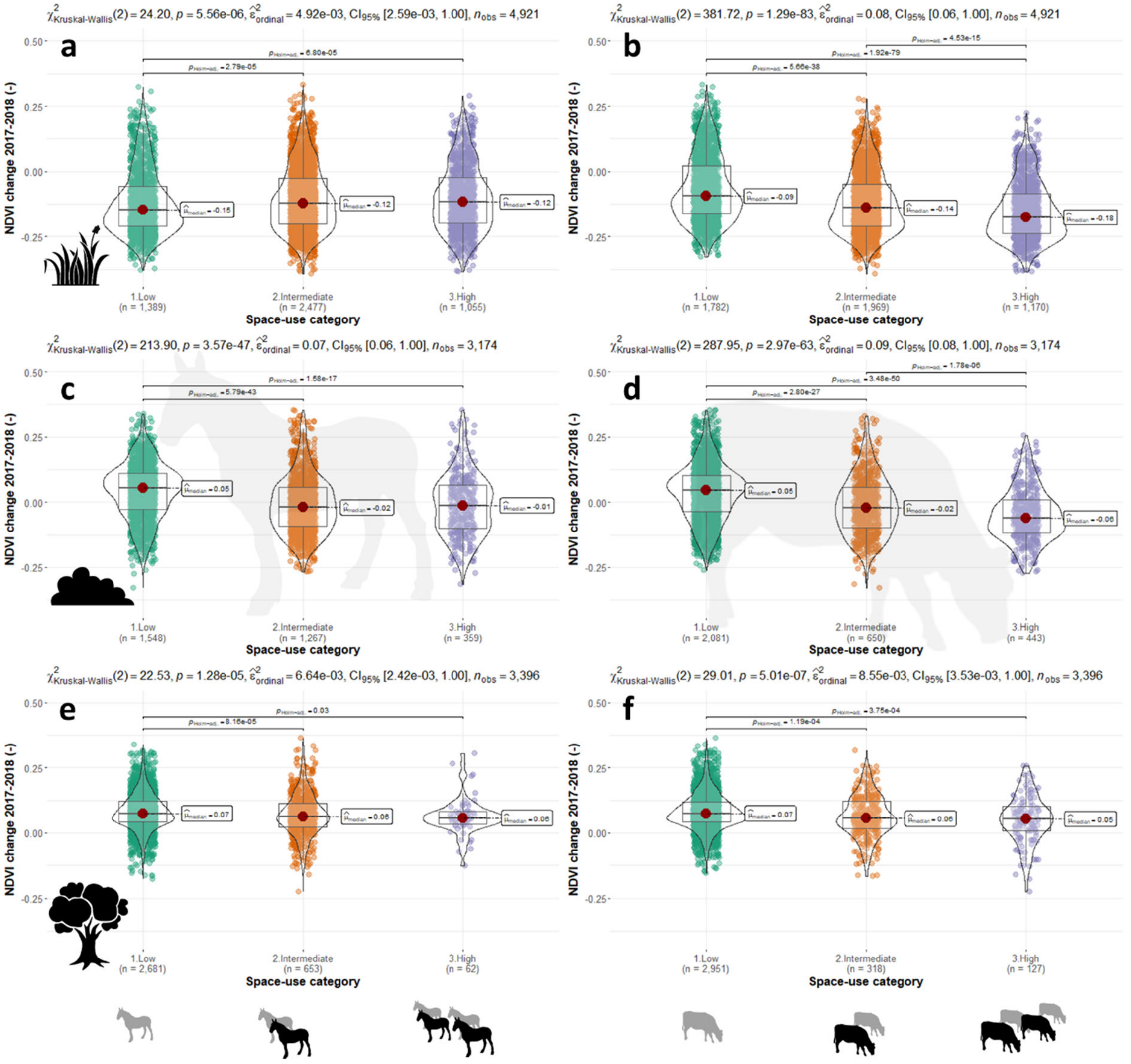

***Figure S13****: NDVI change during the drought (2017-2018) for the three space-use categories (low: <4, intermediate: >4 and <12, high: >12 GPS fix counts per pixel) for horses (a,c,e) and cattle (b,d,f) for short (a,b), intermediate (c,d) and tall (e,f) vegetation. The horizontal line and red dot shows the median, the box edges show quartiles, and the violin plot shows the kernel density distributions of the data. P-values above the plot shows the results of the Kruskal-Wallis test, and the p-values for the pairwise comparisons (Dunn-tests) are shown when significant. The following icons were used under CC BY-3.0 Attribution License from the Noun Project: Grass (Nanik haq), Shrub (wahab marhaban), Tree (kareemov). The following icons were used under CC0 1.0 Universal Public Domain Dedication from Phylopic: Cattle (Bos primigenius taurus Linnaeus 1758), Horse (Equus ferus caballus Linnaeus 1758).*

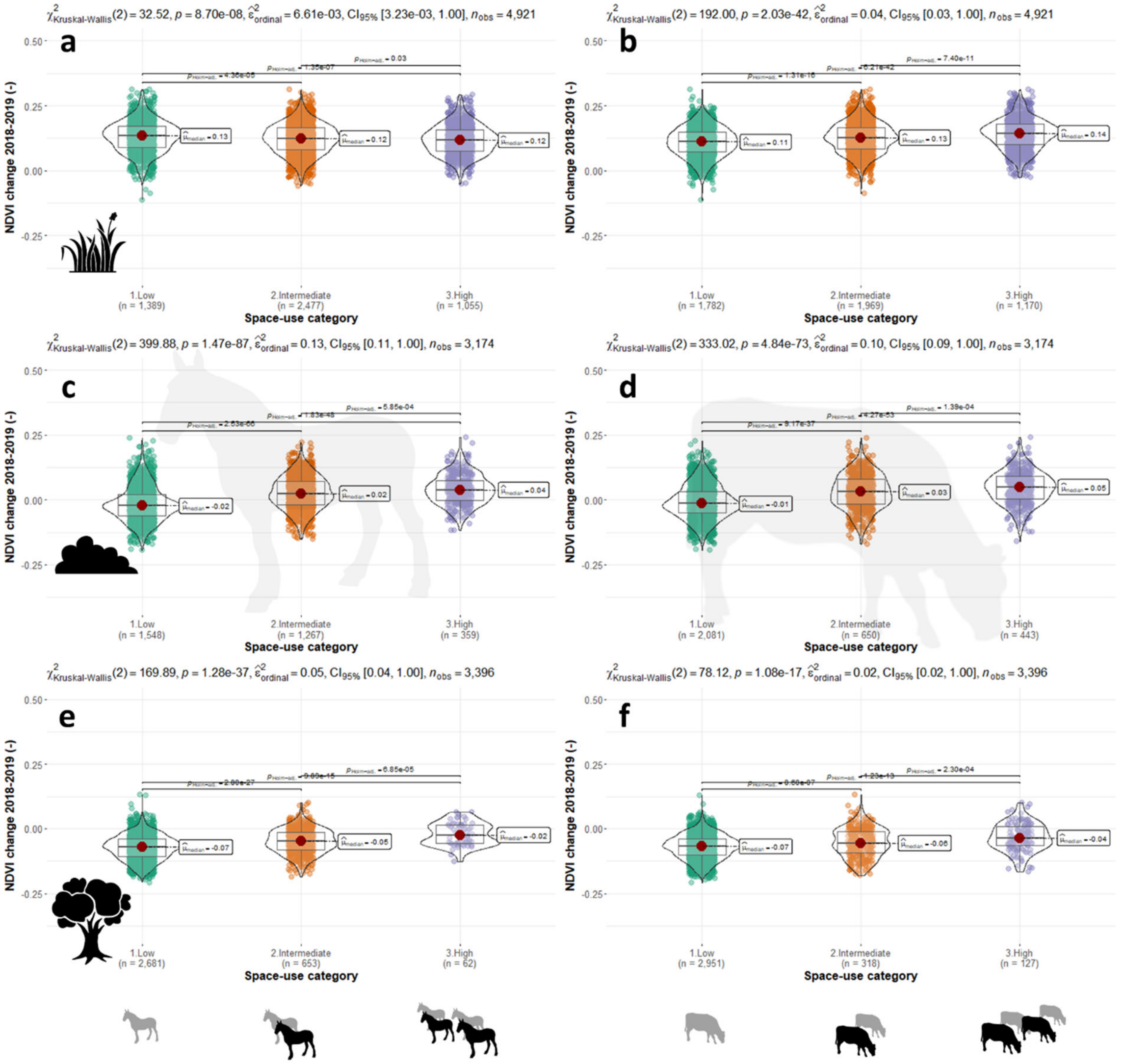

***Figure S14****: NDVI change in the immediate post-drought recovery (2018-2019) for the three space-use categories (low: <4, intermediate: >4 and <12, high: >12 GPS fix counts per pixel) for horses (a,c,e) and cattle (b,d,f) for short (a,b), intermediate (c,d) and tall (e,f) vegetation. The horizontal line and red dot shows the median, the box edges show quartiles, and the violin plot shows the kernel density distributions of the data. P-values above the plot shows the results of the Kruskal-Wallis test, and the p-values for the pairwise comparisons (Dunn-tests) are shown when significant. The following icons were used under CC BY-3.0 Attribution License from the Noun Project: Grass (Nanik haq), Shrub (wahab marhaban), Tree (kareemov). The following icons were used under CC0 1.0 Universal Public Domain Dedication from Phylopic: Cattle (Bos primigenius taurus Linnaeus 1758), Horse (Equus ferus caballus Linnaeus 1758).*

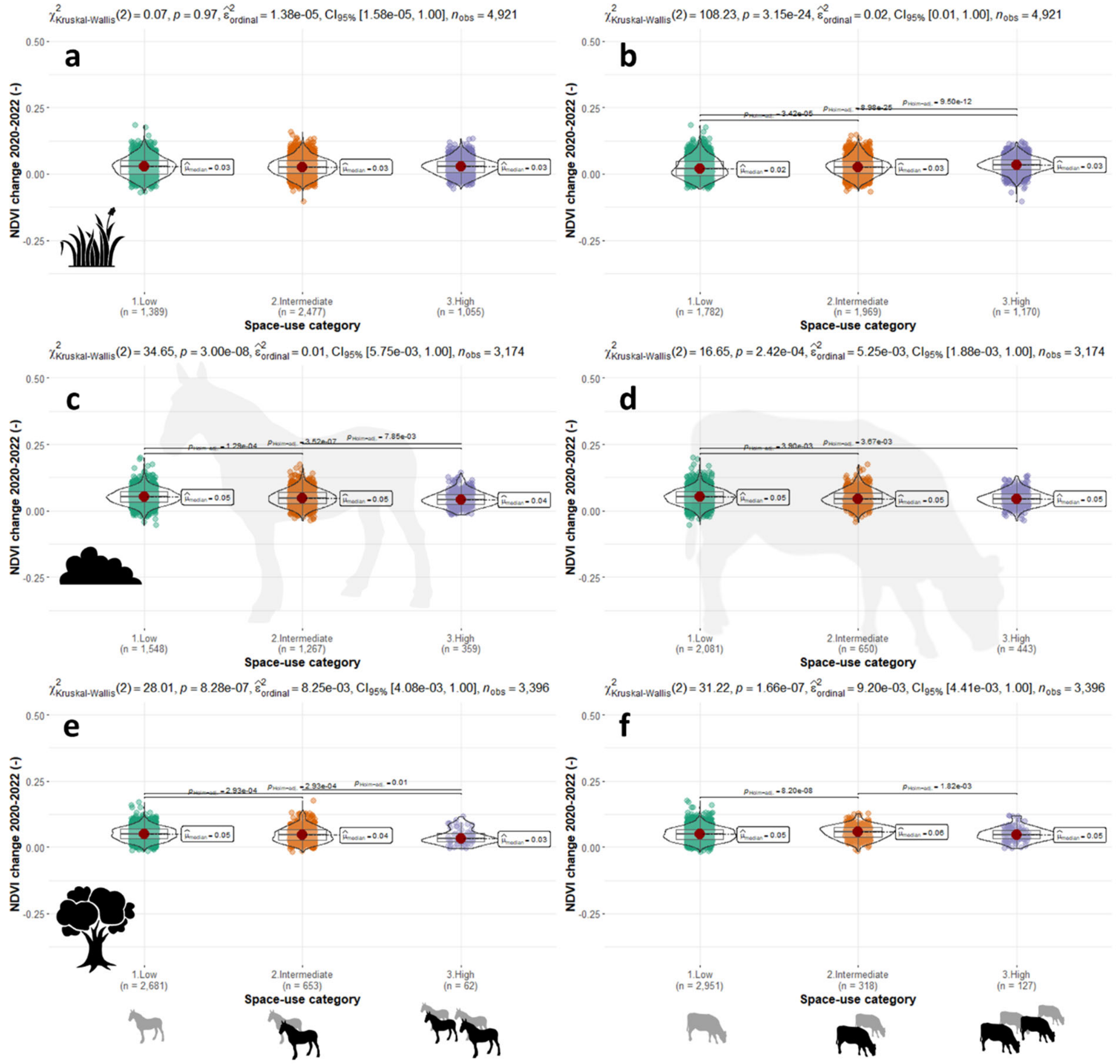

***Figure S15**: NDVI change following the ~2/3 population reduction (2020-2022) for the three space-use categories (low: <4, intermediate: >4 and <12, high: >12 GPS fix counts per pixel) for horses (a,c,e) and cattle (b,d,f) for short (a,b), intermediate (c,d) and tall (e,f) vegetation. The horizontal line and red dot shows the median, the box edges show quartiles, and the violin plot shows the kernel density distributions of the data. P-values above the plot shows the results of the Kruskal-Wallis test, and the p-values for the pairwise comparisons (Dunn-tests) are shown when significant. The following icons were used under CC BY-3.0 Attribution License from the Noun Project: Grass (Nanik haq), Shrub (wahab marhaban), Tree (kareemov). The following icons were used under CC0 1.0 Universal Public Domain Dedication from Phylopic: Cattle (Bos primigenius taurus Linnaeus 1758), Horse (Equus ferus caballus Linnaeus 1758).*

# References

Assmann, J. J., J. E. Moeslund, U. A. Treier, and S. Normand. 2022. EcoDes-DK15: high-resolution ecological descriptors of vegetation and terrain derived from Denmark's national airborne laser scanning data set. Earth System Science Data 14:823–844.

Bonavent, C., K. Olsen, R. Ejrnæs, C. Fløjgaard, M. D. Hansen, S. Normand, J. Svenning, and H. H. Bruun. 2023. Grazing by semi-feral cattle and horses supports plant species richness and uniqueness in grasslands. Applied Vegetation Science 26:e12718.

Craiu, R. V., T. Duchesne, D. Fortin, and S. Baillargeon. 2011. Conditional logistic regression with longitudinal follow-up and individual-level random coefficients: A stable and efficient two-step estimation method. Journal of Computational and Graphical Statistics 20.

Fløjgaard, C., P. B. M. Pedersen, C. J. Sandom, J.-C. Svenning, and R. Ejrnæs. 2022. Exploring a natural baseline for large-herbivore biomass in ecological restoration. Journal of Applied Ecology 59:18–24.

Houmark-Nielsen, M., K. L. Knudsen, and N. Noe-Nygård. 2006. Istider og Mellemistider. Pages 255–302 Naturen i Danmark - Geologien. 1st edition. Gyldendal, Copenhagen.

Jonsen, I. D., M. Basson, S. Bestley, M. V. Bravington, T. A. Patterson, M. W. Pedersen, R. Thomson, U. H. Thygesen, and S. J. Wotherspoon. 2013. State-space models for bio-loggers: A methodological road map. Deep-Sea Research Part II: Topical Studies in Oceanography 88–89.

Jonsen, I. D., J. M. Flemming, and R. A. Myers. 2005. Robust state-space modeling of animal movement data. Ecology 86.

Karelus, D. L., J. Walter McCown, Brian K. Scheick, Madelon van de Kerk, Benjamin M. Bolker, and Madan K. Oli. 2019. Incorporating movement patterns to discern habitat selection: black bears as a case study. Wildlife Research 46:76–88.

Michelot, T., N. J. Klappstein, J. R. Potts, and J. Fieberg. 2024. Understanding step selection analysis through numerical integration. Methods in Ecology and Evolution 15:24–35.

Michelot, T., R. Langrock, and T. A. Patterson. 2016. moveHMM: an R package for the statistical modelling of animal movement data using hidden Markov models. Methods in Ecology and Evolution 7:1308–1315.

Thurfjell, H., S. Ciuti, and M. S. Boyce. 2014. Applications of step-selection functions in ecology and conservation. Movement Ecology 2.

Zucchini, W., I. L. Macdonald, and R. Langrock. 2017. Hidden Markov models for time series: An introduction using R, second edition. Page Hidden Markov Models for Time Series: An Introduction Using R, Second Edition.
